# Chalcone Derivative CX258 Suppresses Colorectal Cancer via Inhibiting the TOP2A/Wnt/β-Catenin Signaling

**DOI:** 10.3390/cells12071066

**Published:** 2023-03-31

**Authors:** Xi Chen, Xiaocheng Lv, Lijie Gao, Jiawei Liu, Wei Wang, Lichao Guo, Mykhaylo S. Frasinyuk, Wen Zhang, David S. Watt, Chunming Liu, Xifu Liu

**Affiliations:** 1 Ministry of Education Key Laboratory of Molecular and Cellular Biology, Hebei Anti-Tumor Molecular Target Technology Innovation Center, College of Life Science, Hebei Normal University, Shijiazhuang 050024, China; 2 Institute of Bioorganic Chemistry and Petrochemistry, National Academy of Science of Ukraine, 02094 Kyiv, Ukraine; 3 Lucille Parker Markey Cancer Center, University of Kentucky, Lexington, KY 40536, USA; 4 Department of Molecular and Cellular Biochemistry, College of Medicine, University of Kentucky, Lexington, KY 40536, USA

**Keywords:** chalcone derivative, colorectal cancer, Wnt/β-catenin signaling, TOP2A, cell cycle, cell proliferation

## Abstract

The deregulation in the Wnt/β-catenin signaling pathway is associated with many human cancers, particularly colorectal cancer (CRC) and, therefore, represents a promising target for drug development. We have screened over 300 semisynthetic and natural compounds using a Wnt reporter assay and identified a family of novel chalcone derivatives (CXs) that inhibited Wnt signaling and CRC cell proliferation. Among them, we selected CX258 for further in vitro and in vivo study to investigate the molecular mechanisms. We found that CX258 significantly inhibited β-catenin expression and nuclear translocation, inducing cell cycle arrest at the G2/M phase in CRC cells. Additionally, CX258 reduced the expression of DNA Topoisomerase II alpha (TOP2A) in CRC cells. Moreover, knocking down TOP2A by siRNAs inhibited the Wnt/β-catenin signaling pathway, a finding suggesting that CX258 inhibited Wnt/β-catenin signaling and CRC cell proliferation at least partially by modulating TOP2A. Further studies showed that CDK1 that interacts with TOP2A was significantly reduced after TOP2A knockdown. We demonstrated that CX258 significantly inhibited DLD-1 CRC cell xenografts in SCID mice. In summary, we identified CX258 as a promising candidate for colorectal cancer treatment by targeting the TOP2A/Wnt/β-catenin signaling pathway.

## 1. Introduction

Colorectal cancer (CRC) is the third most common cancer and a leading cause of cancer-related mortality across the world [1,2,3]. More than two million new diagnoses of colon cancer were made in 2020 alone [1,2,3]. Despite recent advances in treatment, the prognosis for CRC remains poor owing to the high rate of metastases and post-intervention recurrence [4]. The Wnt/β-catenin signaling pathway plays an indispensable role in embryogenesis and tissue homeostasis [5]. Abnormal activation of this pathway is associated with developmental diseases and cancers, and this activation is a key driver of the occurrence and progression of CRC [6,7]. Nuclear accumulation of β-catenin has been observed in up to 80% of patients with CRC. The interaction between nuclear β-catenin and T cell transcription factor 4 (TCF4) coactivates downstream gene transcription that, in turn, drives CRC [8,9]. Many small-molecule Wnt inhibitors have been developed, but most of these inhibitors target upstream Wnt signaling by inducing β-catenin degradation. However, many inhibitors proved to be less effective in CRC cells than expected due to mutations in β-catenin and adenomatous polyposis coli protein (APC) that stabilize β-catenin. More importantly, although there are a number of potent Wnt inhibitors, they have not achieved clinical approval because they exhibited dose-limiting toxicity. As a consequence, it is important to develop new Wnt inhibitors that target downstream of Wnt signaling, such as β-catenin nuclear transportation and its activation in the nucleus. 

Chalcones are a group of natural products with antitumor activity. They have been reported to inhibit multiple stages of tumor occurrence and development [10,11] by regulating the cell cycle, cell proliferation, angiogenesis, and apoptosis [12,13,14,15]. As for many natural products, chalcones mediate cell cycle arrest and cytotoxicity by down-regulating cyclin expression, inhibiting topoisomerase II, or enhancing P21 (CIP1/WAF1) expression [16,17]. Many of these natural products or their derivatives are widely used as anticancer drugs [18,19,20]. Topoisomerase 2-alpha (TOP2A) acts as a key enzyme in DNA replication and transcription by altering the topologic states of DNA [21]. TOP2A is overexpressed in various human malignancies, including primary breast cancer, lung cancer, hepatocellular carcinoma, nasopharyngeal carcinoma, adrenal cortex, and colon cancer, indicating that TOP2A is an important drug target and a potential biomarker for these cancers [22,23,24,25,26,27,28]. For example, the miR-139/TOP2A/β-catenin axis was identified as a biomarker for diagnosis and became a valid therapeutic target for cancer treatment [29]. In this study, we showed that chalcone derivative CX258 was a potent antitumor inhibitor that inhibited CRC cell proliferation in vitro and in vivo by targeting TOP2A/Wnt/β-catenin signaling. The findings reported herein suggest that chalcone analogs may provide a new therapeutic strategy for colon cancer treatment.

## 2. Materials and Methods

### 2.1. Cell Lines and Cell Cultures

HEK293T, LS174T, HCT116, DLD-1, and HT29 cell lines were purchased from the ATCC (ATCC, Manassas, VA, USA). LS174T and DLD-1 colon cancer cells were cultured in RPMI 1640 (Wuhan Xavier Biotechnology Co., Ltd., Wuhan, China) containing 10% (*v*/*v*) fetal bovine serum (Gibco-BRL, Grand Island, NY, USA) and 1% penicillin-streptomycin (Wuhan Xavier Biotechnology Co., Ltd., Wuhan, China). HCT116 and HT29 colon cancer cells and HEK293T cells were grown in Dulbecco’s modified Eagle’s medium (Wuhan Xavier Biotechnology Co., Ltd., Wuhan, China) supplemented with 10% fetal bovine serum and 1% penicillin-streptomycin (Wuhan Xavier Biotechnology Co., Ltd., Wuhan, China). All cells were cultured at 37 °C with 5% CO_2_ in a water-jacketed incubator. Cell viability and number were analyzed using a Vi-CELL XR cell viability analyzer (Beckman Coulter, Indianapolis, IN, USA).

### 2.2. Reporter Assay

HEK293T cells were transfected with a plasmid containing Super 8× TOPFlash subcloned into pGL4.83 (hRlucP/Puro) (provided by Professor Chunming Liu, University of Kentucky). A stable HEK293T cell line containing the TOPFlash reporter was established by puromycin selection. Potential chalcone inhibitors were screened with the stable cell line by measuring firefly luciferase activities (Titertek Berthold, Pforzheim, BW, Germany) after the treatment with DMSO or the testing of compounds in DMSO solution, and LiCl was used to activate Wnt signaling at a final concentration of 2.5 mM. In order to verify if these compounds directly acted on the Wnt/β-catenin/TCF complex, HEK293T cells were transiently transfected with either a TOPFlash or a FOPFlash reporter (Super 8× TOPFlash) and Renilla, followed by co-transfection of Wnt3A, wild-type β-catenin, β-catenin (S33A) mutants, LEF-VP16, respectively (these plasmids were provided by Professor Chunming Liu, University of Kentucky). After 24 h of culture, the cells were treated with CX258 (5 μM) or DMSO as a control. Luciferase activity was determined using a dual-luciferase reporter assay system (Promega Biotechnology Co., Ltd., Madison, WI, USA) using a Sirius-L luminometer (Titertek Berthold, Pforzheim, BW, Germany). Renilla luciferase activity was used as an endogenous control. All experiments were performed at least twice. 

### 2.3. Cell Proliferation Assay

Cells were seeded at a density of 4 × 10^4^ cells per well in 12-well plates and cultured at 37 °C for 24 h, followed by the addition of 1 μL of each compound in DMSO to each well to give a final concentration of 10 μM. DMSO was used as a control. Each experiment was performed in triplicate. After 5 days, the medium was removed, and the cells were digested with 200 µL trypsin (0.25%) for 5 min, resuspended in 800 µL phosphate-buffered saline, and counted using a cell viability analyzer (Beckman Coulter, Indianapolis, IN, USA). IC_50_ data were analyzed by GraphPad Prism 8.0.1, and the parameter was selected as log(inhibitor) vs. response.

### 2.4. Western Blot Assay

The cells were lysed in an appropriate volume of lysis buffer (50 mM HEPES, 100 mM NaCl, 2 mM EDTA, 1% (*v/v*) glycerol, 50 mM NaF, 1 mM Na_3_VO_4_, 1% (*v/v*) Triton X-100, and protease inhibitors) for 20 min on ice. Supernatant was collected after centrifugation at 13,000 rpm for 30 min. Equal amounts of protein were resolved by SDS-polyacrylamide gel electrophoresis and electro-blotted onto equilibrated PVDF membranes (EMD Millipore, Billerica, MA, USA). After blocking in Tris-buffered saline (TBS) containing 5% non-fat milk, the membranes were immunoblotted with primary antibody of target proteins overnight at 4 °C. The antibodies and the dilutions for Western blots used in these studies are as follows: β-catenin (1:1000, Cell Signaling Technology, Danvers, MA, USA, 8480T), Axin2 (1:1000, Cell Signaling Technology, 2151), c-Myc (1:1000, Epitomics, Burlingame, CA, USA, 1472-1), TOP2A (1:1000, Proteintech, Wuhan, China, 20233-1-AP), CDK1 (1:1000, Cell Signaling Technology, 9116T), P53 (1:1000, Cell Signaling Technology, 2527T), P21 (1:1000, Cell Signaling Technology, 2947T), CDK4 (1:1000, Cell Signaling Technology, 12790T), and GAPDH (1:1000, Proteintech, 10494-1-AP) were used in this study. After three washes in TBS containing 0.1% Tween 20 (TBST), membranes were incubated with HRP-conjugated antibodies. The following products were used in these experiments: goat anti-rabbit IgG (H + L) secondary antibody (1:10,000, Thermo Fisher Scientific, Waltham, MA, USA, 31460), HRP-linked anti-mouse IgG (1:10,000, Cell Signaling Technology, 7076S). Membranes were subsequently incubated with ECL-enhanced chemiluminescence reagent (ZETA Life Inc., New York, NY, USA) for 1–2 min and exposed to the Bio-Rad ChemiDoc™ XRS System (BioRad Laboratories, Inc., Hercules, CA, USA).

### 2.5. Cell Cycle Assay

Cells were plated at a density of 5 × 10^5^ cells per well in 12-well plates and cultured at 37 °C for 24 h, followed by the addition of testing CX258 at concentrations of 1, 3, 5, and 10 μM, respectively. Treated cells were cultured for another 24 h. The cells were trypsinized (0.25% trypsin) for 5 min and collected by centrifugation. According to the kit (Hangzhou Lianke Biotechnology Co., Ltd., Hangzhou, China) instructions, the permeabilization solution (10 μL) and DNA staining solution (1 mL) were added, vortexed for 5–10 s, mixed evenly, and stained for 30 min in the dark. Cells were then sorted by fluorescence-activated cell sorter scan (FACS, CytoFLEX, Beckman Coulter, Brea, CA, USA), according to the manufacturer’s instructions (emission wavelength: 615 nm; excitation wavelength: 535 nm). The percentages of cells in each cell cycle phase were analyzed by ModFit LT™ 3.1 software (VeritySoftwareHouse, Topsham, ME, USA). Each experiment was performed in triplicate. 

### 2.6. Immunofluorescence

Cells were seeded at a density of 4 × 10^4^ cells/well on slides in 24-well plates and cultured at 37 °C for 24 h, followed by the addition of CX258 in DMSO to each well to give a final concentration of 5 μM. These cells were cultured for another 24 h. The slides used to analyze these lysed cells were washed with PBS, fixed in 4% paraformaldehyde for 10 min, washed again with PBS, and permeabilized with 0.5% Triton X-100. Each well was blocked for 30 min with 10% bovine serum albumin (BSA). The primary antibody (β-catenin, 1:200, Cell Signaling Technology, 8480T) was diluted in PBS containing 1% bovine serum albumin and incubated overnight at 4 °C. The secondary antibody (Goat Anti-Rabbit IgG H&L/AF488 antibody, 1:500, Bioss, Boston, MA, USA, bs-0295G-AF488) was diluted with 1% bovine serum albumin and incubated for 2 h at 25 °C. DAPI staining (Servicebio Biotechnology Co., Ltd., Wuhan, China) was performed for 15 min. Images were captured using an ImageXpress Micro Confocal microscope (Molecular Devices, LLC, Sunnyvale, CA, USA).

### 2.7. RNA-Seq

RNA-Seq was performed to evaluate the differential gene expression profiles of LS174T cells treated with 5 μM CX258 or DMSO for 24 h, and the significantly dysregulated mRNA levels were verified by qRT-PCR. The Kyoto Encyclopaedia of Genes and Genomes (KEGG) pathway enrichment analysis was used to predict the potential mechanisms of action of CX258 in colon cancer cells. The functional protein association network was constructed by STRING (https://string-db.org/cgi/input.pl (accessed on 2 April 2022)) [30]. 

### 2.8. Quantitative RT-PCR

Cells were seeded at 1 × 10^6^ cells/well in 6-well plates and treated with DMSO or inhibitors in DMSO. After 24 h, the cells were collected, and total RNA was extracted using the Eastep^®^ Super Total RNA extraction kit (Promega Biotechnology Co., Ltd., Madison, WI, USA). cDNA was synthesized from 1 μg of total RNA using a Reverse Transcription Kit (Thermo Fisher Scientific, formerly of Savant, MA, USA), according to the manufacturer’s instructions. Quantitative reverse transcription PCR (qRT-PCR) was performed using ChamQ SYBR qPCR Master Mix (Norvezan Biotechnology Co., Ltd., Nanjing, China) on a real-time PCR system (CFX 96 Real-Time PCR Detection System, BioRad Laboratories, Inc., Hercules, CA, USA). Thermal cycling was conducted at 95 °C for 10 min, followed by 40 cycles of amplification at 95 °C for 15 s and then at 60 °C for 1 min. The melting program was as follows: 95 °C for 15 s, 60 °C for 1 min, and 95 °C for 15 s. Relative quantification of gene expression in each sample was analyzed using the 2^−ΔΔCt^ method. Each experiment was performed in triplicate. We designed specific primers for TOP2A (forward: 5′-CATTGAAGACGCTTCGTT-ATGG-3′; reverse 5′-CCAGTTGTGATGGATAAAATTAATCAG-3′), CDK1 (forward: 5′-AAACTACAGGTCAAGTGGTAGCC-3′; reverse 5′-TCCTGCATAAGCACATCCTGA-3′), and GAPDH (forward: 5′-TGTGGGCATCAATGGATTTGG-3′; reverse 5′-ACACCATGTATTCCGGGTCAAT-3′).

### 2.9. Small Interfering RNA (siRNA) Transfection

siRNA-TOP2A was designed and synthesized (Sangon Biotech, Shanghai, China) in order to knock down TOP2A expression. Transfection was performed using Lipofectamine 2000 (Invitrogen, Carlsbad, CA, USA), according to the manufacturer’s instructions. 

The sequences of the siRNAs: 

si-TOP2A-T765 (sense: 5′-GCCUGAUUUGUCUAAGUUUAATT-3′; antisense: 5′-UUAAACUUAGACAAAUCAGGCTT-3′).

si-TOP2A-T2698 (sense: 5′-CCCAACUUUGAUGUGCGUGAATT-3′; antisense: 5′-UUCACGCACAUCAAAGUUGGGTT-3′).

si-TOP2A-T3476 (sense: 5′-CCUUCAACUAUCUUCUUGAUATT-3′; antisense: 5′-UAUCAAGAAGAUAGUUGAAGGTT-3′).

### 2.10. In-Vivo Evaluation of Anticancer Activity in DLD-1 Mouse Xenografts

NOD/SCID mice (6–8 weeks old) were purchased from the Beijing Vital River Laboratory Animal Technology Co., Ltd. (Beijing, China), housed under specific pathogen-free (SPF) conditions (constant temperature, 25 ± 1 °C; relative humidity, 40–60%; 12-h light/dark cycle), and allowed free access to food and water during the study period. DLD-1 cells in the exponential growth phase were trypsinized and resuspended in 50% Matrigel (Corning Costar, Cambridge, MA, USA). Next, 2 × 10^6^ cells were subcutaneously injected into the back of NOD/SCID mice (2 tumors per mouse). When the subcutaneous tumor volume reached 5 × 5 mm^3^, the mice were divided into a control group and a treatment group (with 75 mg/kg CX258). Every day, CX258 was intraperitoneally (i.p.) administered to the treatment group at a dose of 75 mg/kg body weight. Tumor volumes were measured and recorded every 3 days. Mouse weights were measured twice a week, and the experiment was stopped on day 15. At the end of the experiment, the mice were euthanized. Tumors were isolated, weighed and fixed in 10% neutral-buffered formalin for IHC analysis.

### 2.11. IHC Analysis

Tissue sections were dewaxed, rehydrated, and heated in a microwave in sodium citrate buffer (10 mM, pH 6) for antigen extraction. The tissue slices were incubated with 3% H_2_O_2_ peroxide solution in the dark to block endogenous peroxidase. Goat antiserum was used for blocking at 25 °C for 30 min. The sections were incubated with primary antibodies (Ki-67, 1: 200, Cell Signaling Technology, 9449) to detect the expression levels of target proteins in the tissues. The tissue slices were incubated overnight at 4 °C and washed thoroughly with PBS. The secondary antibody (Zhongshanjinqiao SP9000) was incubated at 25 °C for 1 h. A DAB chromogenic solution (Zhongshanjinqiao Biotechnology Co., Ltd., Beijing, China) was used to stain the cells. The sections were placed in hematoxylin solution to restore the nucleus. The samples were then dehydrated, sealed, observed and photographed under a microscope.

### 2.12. Statistics

Data are presented as mean ± SD. Statistical significance across the groups was calculated using Student’s two-tailed *t*-test and one-way ANOVA, and *p* < 0.05 was considered statistically significant. Statistical significance is represented by * if *p* < 0.05, ** if *p* < 0.01, and *** if *p* < 0.001.

## 3. Results

### 3.1. Identification of Novel Chalcone Inhibitors of Wnt Signaling through TOPFlash Screening Assay

**Figure 1:**
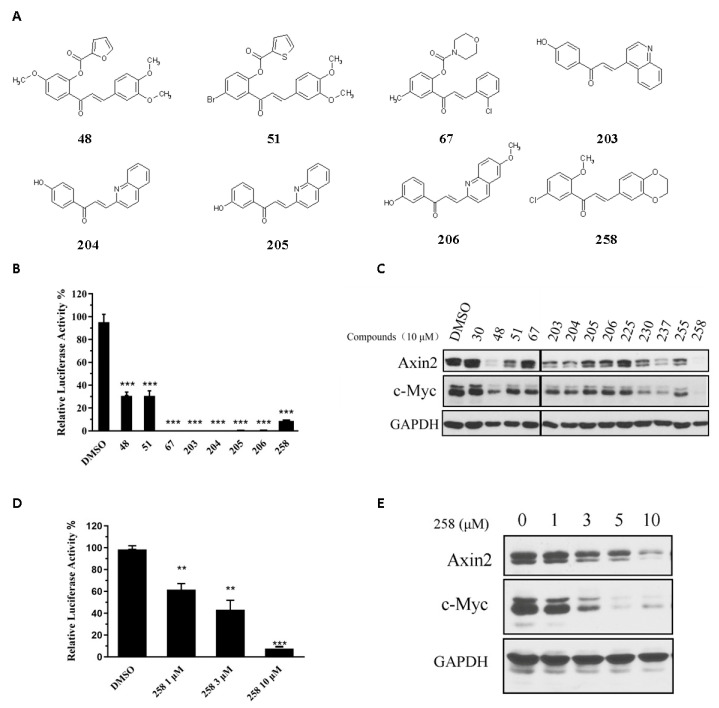
Identification of chalcone inhibitors of Wnt signaling through TOPFlash screening assay. (**A**) Chemical structure of selected chalcone derivatives. (**B**) Chalcone derivatives (10 μM) inhibited TOPFlash reporter activation. *** *p* < 0.001 vs. DMSO group. (**C**) Chalcone derivatives (10 μM) reduced the protein levels of Wnt signaling pathway target genes Axin2 and c-Myc in LS174T cells. GAPDH was used as a loading control. (**D**) TOPFlash reporter assay with different concentrations of CX258 for 24 h. ** *p* < 0.01, *** *p* < 0.001. (**E**) Western blot identified the inhibitory effect of different concentrations of CX258 on Axin2 and c-Myc protein levels in LS174T cells. GAPDH was used as a loading control.

We screened 300+ synthetic, semisynthetic or naturally occurring chalcones using a Wnt reporter assay. We identified a family of chalcone derivatives that inhibited Wnt signaling reporter and Wnt signaling downstream genes, including c-Myc and Axin2 (Figure 1A–C). However, these chalcone analogs did not inhibit the activity of a FOPFlash reporter that was used as a negative control of a TOPFlash reporter, a finding that suggested that these chalcone derivatives inhibited the Wnt signaling pathway (Figure S1). Synthesis and definitive structural characterization of selected chalcone derivatives (Figure 1C) are described in Supplementary File S1. Based on the inhibition effects on endogenous Wnt target genes (Figure 1C), we identified chalcone derivative CX258 as the leading candidate for further studies. CX258 decreased TOPFlash reporter activity induced by either Wnt3A and LiCl in a concentration-dependent manner (Figure 1D and Figure S2). In addition, CX258 inhibited the expression of Wnt target genes in CRC cells at both mRNA and protein levels (Figure 1E and Figure S3).

### 3.2. CX258-Induced G2/M Cell Cycle Arrest and Inhibited CRC Cell Proliferation

**Figure 2:**
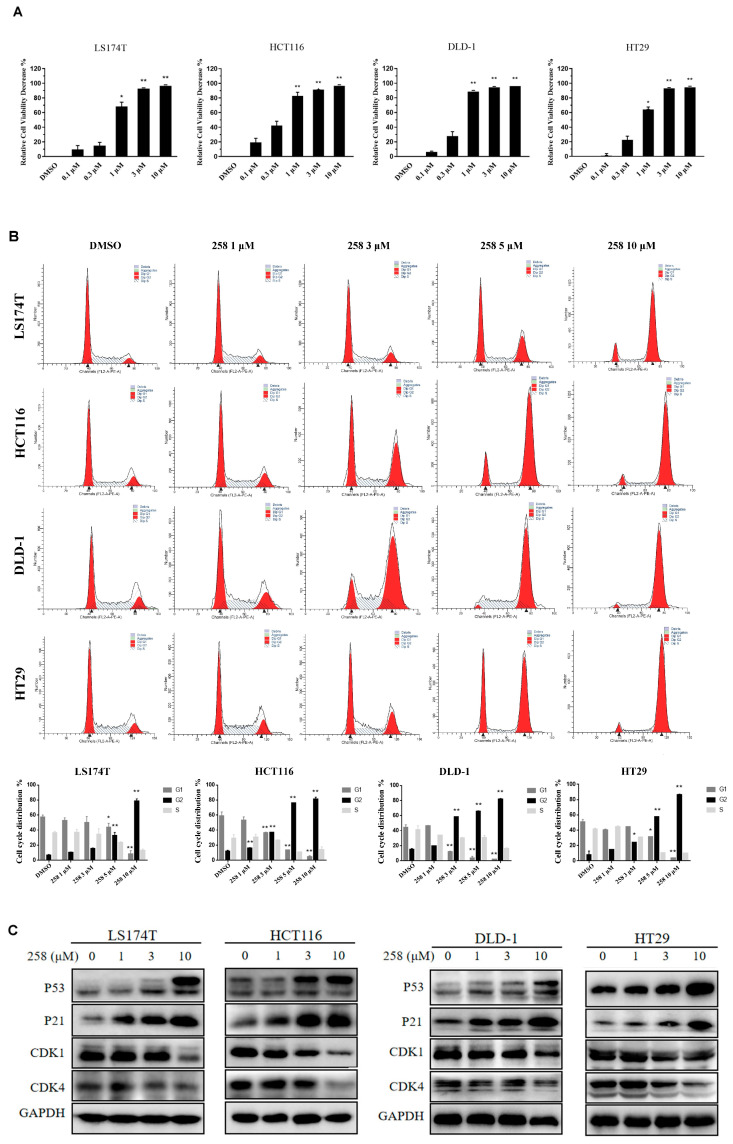
CX258 induced cell cycle arrest at the G2/M phase and inhibited CRC cell proliferation. (**A**) CX258 inhibited the proliferation of LS174T, HCT116, DLD-1 and HT29 CRC cells. Data are presented as the mean ± SD. * *p* < 0.05, ** *p* < 0.01. (**B**) CX258 induced the cell cycle arrest of CRC cells at the G2/M phase. The cell cycles were analyzed by PI staining and flow cytometry. Data are presented as the mean ± SD. * *p* < 0.05, ** *p* < 0.01. (**C**) CX258 induced P53 and P21 and inhibited CDK1 and CDK4 in CRC cells. The cells were treated with CX258 or DMSO for 24 h. GAPDH was used as a loading control.

The chalcone CX258 inhibited in vitro cell growth of well-known CRC cell lines: LS174T (IC_50_ = 0.42 ± 0.01 μM), HCT116 (IC_50_ = 0.34 ± 0.09 μM), HT29 (IC_50_ = 0.65 ± 0.08 μM) and DLD-1 (IC_50_ = 0.32 ± 0.04 μM) (Figure 2A). CX258 was more potent than naturally occurring chalcones on tumor cell inhibition (Figure S4). The effect of CX258 on the CRC cell cycle was analyzed by PI staining and flow cytometry. CX258 blocked the cell cycle at the G2/M phase (Figure 2B). In addition, the expression of tumor suppressor genes P53 and P21 was increased. Meanwhile, the expression of CDK1 and CDK4 decreased in a dose-dependent manner after CX258 treatment (Figure 2C). P53 is a tumor suppressor gene that negatively regulates the cell cycle. P21 occurs downstream of P53, constitutes the G1 checkpoint of the cell cycle, and inhibits cell cycle progression [31]. CDK1 and CDK4 are directly involved in the regulation of the cell cycle; CDK1 mainly plays a role in the G2/M phase, and CDK4 plays a role in the G1 phase. The cyclin/CDK complex promotes the transformation of each restriction point of the cell [32,33]. The cell was pushed to complete the transformation process of each period. These results indicated that CX258 inhibited cell cycle progression that, in turn, inhibited CRC growth.

### 3.3. CX258 Reduced Nuclear β-Catenin Levels and Wnt Target Genes in CRC Cells

**Figure 3:**
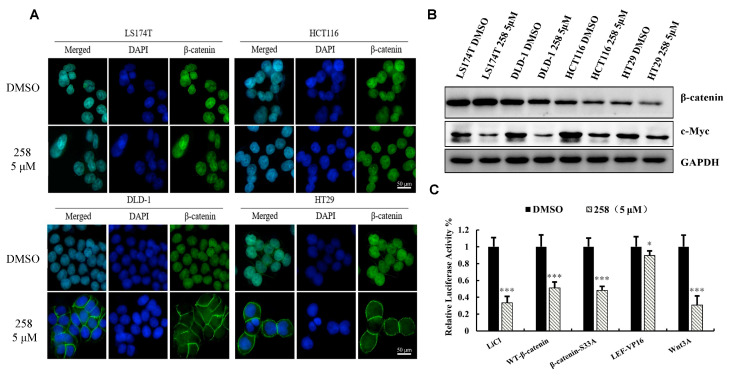
CX258 reduced nuclear β-catenin levels and Wnt target genes in CRC cells. (**A**) CRC cells were analyzed by β-catenin and DAPI immunocytochemical staining. CX258 reduced nuclear β-catenin levels in DLD-1 and HT29 cells (magnification 400×). (**B**) Effects on CX258 on the protein levels of β-catenin and c-Myc. GAPDH was used as the loading control. (**C**) Wnt Reporter assays. CX258 inhibited TOPFlash reporter activities induced by Wnt3A, wild-type β-catenin and β-catenin (S33A) but was less effective in inhibiting the TOPFlash reporter induced by LEF-VP16 fusion protein. * *p* < 0.05, *** *p* < 0.001.

CRC cell lines LS174T, DLD-1, HCT116, and HT29 were treated with DMSO or CX258 (5 μM), and immunofluorescence cytochemistry was performed to examine β-catenin expression and location. CX258 reduced β-catenin levels in the nucleus of DLD-1 and HT-29 cells but not in either LS174T or HCT116 cells (Figure 3A). We also analyzed β-catenin protein levels by western blotting. CX258 slightly reduced the total β-catenin levels in DLD-1 and HCT116 cells but not in either LS174T or HT29 cells. The expression of the Wnt target gene c-Myc was decreased in these CRC cells (Figure 3B). These results suggest that CX258 may regulate Wnt signaling by multiple mechanisms in addition to reducing β-catenin protein levels and nuclear localization.

To investigate the mechanisms of CX258 on Wnt/β-catenin signaling, we performed several TOPFlash reporter assays by activating Wnt signaling with Wnt3A, wild-type β-catenin, β-catenin (S33A) mutants, and the LEF-VP16 fusion protein, respectively. We found that CX258 inhibited Wnt3A, wild-type β-catenin and β-catenin (S33A) induced TOPFlash reporter activities, but CX258 was less effective for LEF-VP16 fusion protein-induced TOPFlash reporter activity (Figure 3C). This finding suggested that CX258 also inhibited Wnt signaling at transcription levels mediated by β-catenin/TCF.

### 3.4. The Effects of CX258 on Transcriptome of CRC Cells

**Figure 4:**
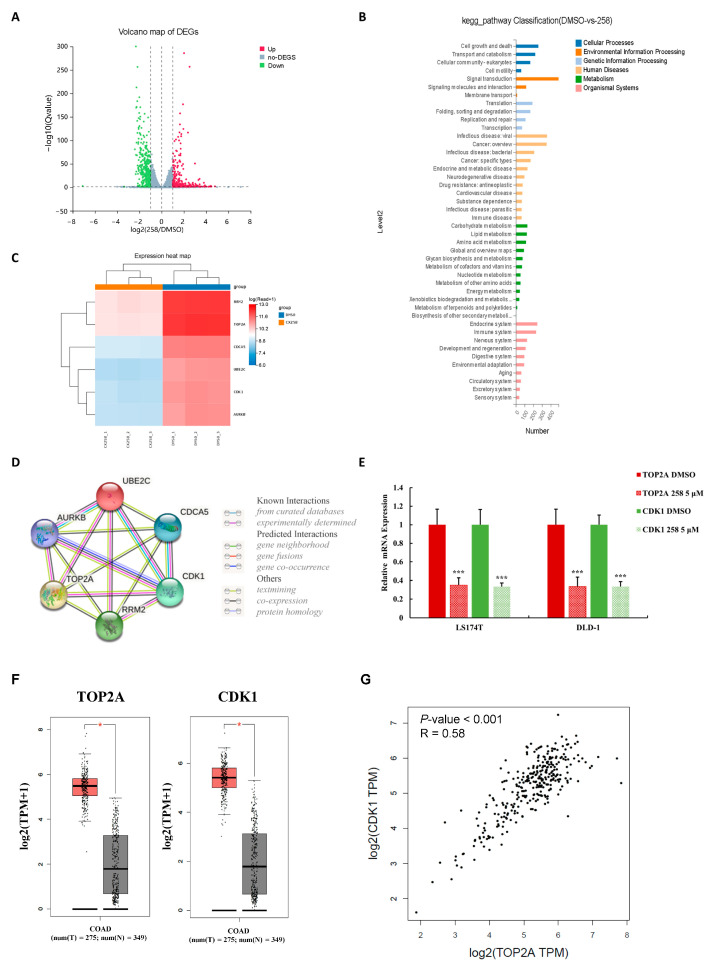
The effects of CX258 on the transcriptome of CRC cells. (**A**) Volcano map of differentially expressed genes (DEGs), including 2306 up-regulated and 2607 down-regulated mRNA. (**B**) KEGG pathway enrichment analysis of DEGs. (**C**) Heat map matrix of six selected DEGs. (**D**) Protein–protein interaction (PPI) network analysis of selected DEGs. (**E**) Quantitative RT-PCR (qRT-PCR) analysis. CX258 inhibited TOP2A and CDK1 mRNA expression in LS174T and DLD-1 cells. *** *p* < 0.001. (**F**) TOP2A and CDK1 were significantly overexpressed in CRC. TCGA colon adenocarcinoma tissue data (n = 275) and matched TCGA normal and GTEx data (n = 349) were analyzed using the GEPIA program (* *p* < 0.05). (**G**) Correlation of TOP2A and CDK1 expression.

LS174T cells were treated with CX258, and the gene expression profile was analyzed by RNA-Seq (Table S1). A total of 4913 differentially expressed genes (DEGs) were identified, including 2306 up-regulated mRNA and 2607 down-regulated mRNA, compared with the DMSO-treated samples (Figure 4A). KEGG analysis revealed that many cancer-related genes involved in signaling transduction, cell growth and apoptosis were differentially expressed in CX258-treated CRC cells (Figure 4B). Six significantly downregulated genes (RRM2, TOP2A, CDCA5, UBE2C, CDK1, AURKB) were selected (Figure 4C). Using the STRING functional protein association network, interactions between the proteins encoded by these six genes were evaluated from many aspects (Figure 4D). We selected TOP2A and CDK1 for further validation. These two genes were significantly downregulated in CX258-treated CRC cells (Figure 4C). The results were verified using qRT-PCR (Figure 4E). We analyzed the TCGA database using the GEPIA program [34]; the expression levels of TOP2A and CDK1 were significantly increased in CRC (Figure 4F). We also performed a co-expression analysis of clinical specimens in the ONCOMINE database; the expression levels of CDK1 and TOP2A were highly correlated in CRC (Figure 4G). These findings suggested that TOP2A and CDK1 play important roles in CX258-induced CRC cell inhibition.

### 3.5. TOP2A Regulates CDK1 Expression and Wnt/β-Catenin Signaling in CRC Cells

**Figure 5:**
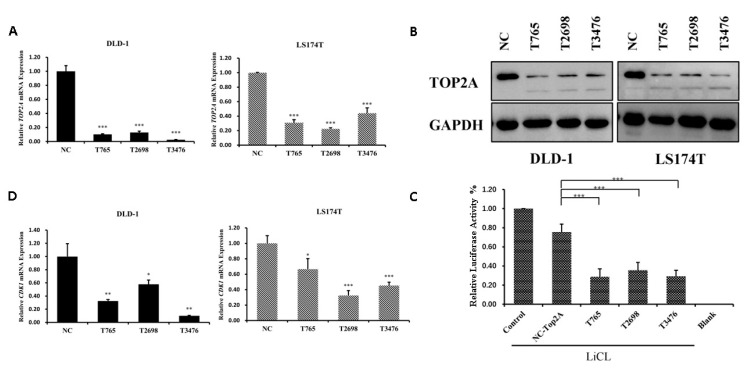
TOP2A regulates CDK1 expression and Wnt/β-catenin signaling in CRC cells. (**A**) Knocking down of TOP2A by siRNAs. The mRNA levels of TOP2A were analyzed by real-time quantitative PCR (qRT-PCR). *** *p* < 0.001 vs. negative control. (**B**) Protein levels of TOP2A were analyzed by western blot. GAPDH was used as a loading control. (**C**) Depletion of TOP2A inhibited TOPFlash reporter activity induced by LiCl. “Control” indicates TOPFlash (800 ng/well) and Renilla vector (200 ng/well) plasmid transfection. “NC-TOP2A” indicates transfection of TOPFlash and Renilla vector plasmid as well as siRNA with low sequence homology and sequences unrelated to the target gene. “T765, T2698 and T3476” indicates transfection of TOPFlash and Renilla vector plasmid as well as siRNA with TOP2A. “Blank” indicates transfection of TOPFlash (800 ng/well) and Renilla vector (200 ng/well) plasmid without LiCl to activate Wnt signaling. *** *p* < 0.001 vs. NC-TOP2A. (**D**) qRT-PCR analysis of the effects of TOP2A siRNAs on the expression of CDK1 in DLD-1 and LS174T cells. * *p* < 0.05, ** *p* < 0.01, *** *p* < 0.001.

To test the effects of TOP2A on Wnt/β-catenin signaling, we knocked down TOP2A in DLD-1 and LS174T cells by siRNAs. Three siRNAs specifically targeting TOP2A (T765, T2698, and T3476) were transfected into DLD-1 and LS174T cells. The silencing effects were confirmed by qRT-PCR and western blot analyses (Figure 5A,B). Next, the TOPFlash reporter gene and siRNAs were co-transfected into HEK-293T cells to verify if the reduction in TOP2A could affect Wnt signaling. Knocking down of TOP2A decreased the activity of luciferase activity induced by the GSK-3 inhibitor, LiCl (Figure 5C). In addition, the silencing of TOP2A reduced the expression of CDK1 (Figure 5D). The results suggested that TOP2A regulated Wnt signaling and cell cycle-related genes. Since CX258 inhibited the expression of TOP2A in CRC cells (Figure 4E), the knockdown of TOP2A reduced the sensitivity of DLD-1 cells to CX258 treatment (Figure S5). CX258 inhibited Wnt signaling and induced cell cycle arrest partially through its effects on TOP2A.

### 3.6. In Vivo Effects of CX258 on CRC Cell Xenografts in SCID Mice

**Figure 6:**
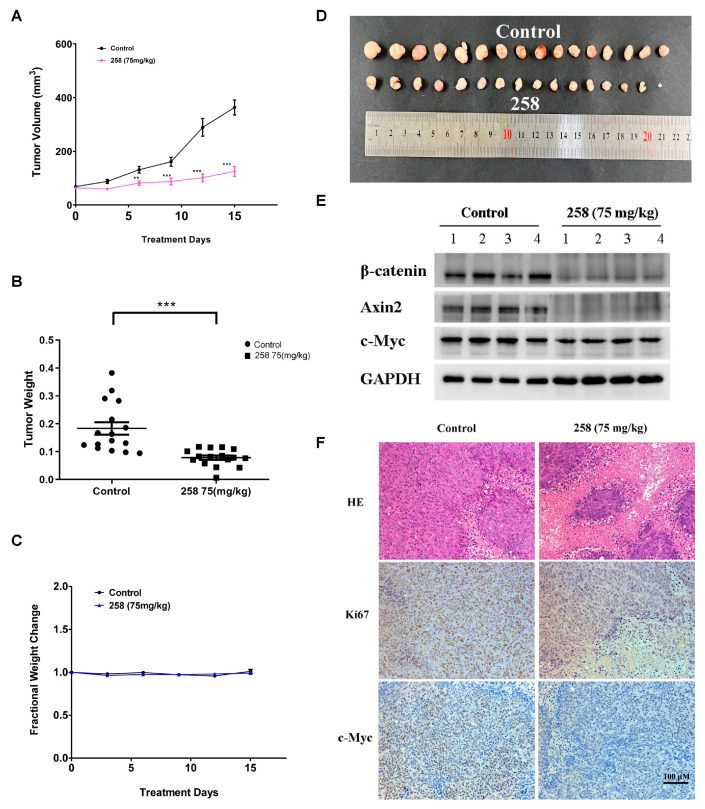
In vivo effects of CX258 on CRC cell xenografts in SCID mice. (**A**–**C**) The chalcone derivative CX258 inhibited DLD-1 xenograft growth in SCID mice without overall toxic effects in terms of body weight. ** *p* < 0.01, *** *p* < 0.001 vs. Control. (**D**) Tumors after 2-week treatment. (**E**) Protein levels of Wnt signaling pathway-related proteins were analyzed by western blot. GAPDH was used as a loading control. (**F**) H&E, Ki67 and c-Myc staining of tumor sections.

To evaluate the tumor-suppressive potential of CX258 in vivo, an NOD/SCID mouse model of colon cancer transplanted with DLD-1 cells was established. CX258 was administrated intraperitoneally (i.p.) at a dosage of 75 mg/kg/day for two weeks, at which point the tumors reached a size of 5 × 5 mm^3^. Tumor volumes and vital signs were monitored and recorded daily. As shown in Figure 6A,B,D, administration of CX258 significantly reduced both tumor growth and tumor weight. Based on the body weight growth rate of the mice, no significant toxicity was observed within 14 days (Figure 6C). The protein levels of β-catenin, Axin2 and c-Myc were significantly reduced in CX258-treated tumors (Figure 6E). Tumor sections were also analyzed using H&E, Ki-67 and c-Myc staining. The cell proliferation marker Ki-67 and Wnt target gene c-Myc were significantly reduced in CX258-treated tumor specimens (Figure 6F).

## 4. Discussion

CRC is one of the leading causes of cancer-related deaths in the world. Almost all types of CRC are characterized by an unusually active Wnt/β-catenin pathway, which is considered a critical cancer initiation and driver event and an important target for CRC treatment [35,36]. Chalcone is an important pharmacophore, and many chalcone derivatives have anticancer activities [37,38]. In this study, we identified the chalcone derivative CX258 (Figure 1A), which was more potent in CRC cell inhibition than the naturally occurring chalcones (Figure S4). Mechanistically, CX258 inhibited Wnt reporter activity and inhibited the expression of Wnt target genes in a concentration-dependent manner (Figure 1D,E). Immunofluorescence and western blot analyses suggested that CX258 inhibited β-catenin expression and nuclear translocation in a subset of CRC cells, thus inhibiting the activation of the Wnt/β-catenin signaling pathway (Figure 3A,B). In general, the β-catenin function can be regulated at multiple steps, including the regulation of β-catenin levels, nuclear localization, β-catenin/TCF interactions, and the interactions of β-catenin/TCF with other transcription regulators [9,39]. In CRC cells, impaired β-catenin degradation results in the accumulation and translocation of cytoplasmic β-catenin to the nucleus. Nuclear β-catenin interacts with T-cell factor 4 (TCF4) and other co-activators, resulting in β-catenin/TCF 4-dependent transcriptional activation of many oncogenes [40,41]. In more than half of all cancer cases, including colorectal carcinoma, liver carcinoma and breast cancer, β-catenin accumulates within the nucleus or cytoplasm [42,43,44]. Therefore, understanding the mechanism of CX258 in Wnt signaling inhibition may lead to potential new agents for CRC treatment. 

In addition, CX258 blocked the cell cycle at the G2/M phase and inhibited CRC cell proliferation (Figure 2A–C). The mammalian cell cycle is induced by successive activation or inactivation of proteins that regulate various stages of the cell cycle. Loss of the normal cell cycle is a well-known hallmark of human cancer. The chalcone derivative CX258 uregulated the protein levels of P53 and P21, downregulated the protein levels of CDK1 and CDK4, inhibited cell cycle progression, and inhibited cell proliferation. In RNA-Seq and KEGG pathway analyses, we found that TOP2A was highly expressed in CRC cells and significantly downregulated by CX258 treatments (Figure 4E,F), an outcome suggesting a potential mechanism of action for CX258. The accumulation of mutations in tumor cells leads to genomic instability and unplanned proliferation. Sister chromatid cohesion is essential for the proper co-segregation of newly duplicated chromosomes at anaphase. TOP2A is involved in DNA damage and repair processes in response to anticancer drugs, environmental factors, or reactive metabolites. Thus, TOP2A plays an important role in regulating genomic stability, and its dysfunction leads to chromosomal rearrangements and cancer, and therefore, it is not surprising that TOP2A is a target for various anticancer drugs [27,45,46]. We found that TOP2A knockdown significantly inhibited the Wnt reporter activity (Figure 5C). Furthermore, CDK1, which interacts with TOP2A, was significantly reduced by TOP2A depletion (Figure 5D). These data suggest that TOP2A acts as a potential mediator for CX258 in inhibiting Wnt/β-catenin signaling and cell cycles in CRC cells. Previous studies have shown that TOP2A mediates pancreatic carcinogenesis by modulating the Wnt/β-catenin pathway [29,47]. However, additional mechanistic studies are required to identify the direct targets of CX258 and to understand the role of TOP2A in Wnt signaling and in cell cycle regulation in CRC cells. Finally, because CX258 inhibited not only key cancer cell lines in various in vitro studies but also inhibited DLD-1 CRC xenografts in SCID mice, chalcones, in general, and specifically, chalcones directly related to CX258, represent a new pharmacophore for future anticancer drug development.

## 5. Conclusions

**Figure 7:**
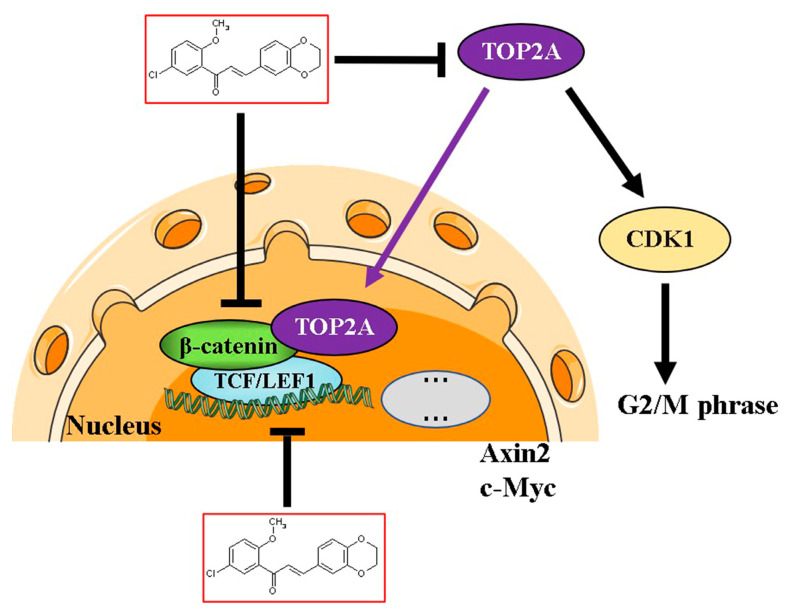
Potential mechanism of action. The chalcone derivative CX258 may inhibit the Wnt signaling pathway by modulating β-catenin levels, nuclear translocation and β-catenin’s activity in Wnt target gene transcription. In addition, CX258 inhibited the expression of TOP2A, reduced the expression of CDK1, induced cell cycle arrest of CRC cells at the G2/M phase, and ultimately inhibited CRC growth.

In summary, we identified CX258 as a novel inhibitor of the TOP2A/Wnt/β-catenin signaling (Figure 7). TOP2A is a potential mediator of the Wnt signaling and cell cycle regulation. Additional studies of chalcones may lead to new therapeutic strategies and agents for CRC treatment.

## 6. Patents

In accord with the University of Kentucky policy, the information in this publication was disclosed to the Office of Technology Commercialization, and a provisional patent (Appl. No. 63/408,657) was jointly filed by the University of Kentucky Research Foundation and Hebei Normal University.

## Data Availability

RNA-seq analysis data have been provided in the Supplementary Material, and raw sequencing data are available upon request. Please contact the corresponding authors for all data requests.

## Supplementary Materials

The following supporting information can be downloaded at: https://www.mdpi.com/article/10.3390/cells12071066/s1, Supplementary File S1: Supplementary methods for chemical synthesis of chalcone derivatives; Supplementary Figure S1: Chalcone derivatives did not inhibited FOPFlash reporter activation; Figure S2: CX258 significantly inhibited TOPFlash activity; Figure S3: CX258 inhibited Wnt target gene transcription in LS174T and DLD-1 cells; Figure S4: Natural chalcones inhibited the proliferation of colon cancer cells; Figure S5: Knocking down of TOP2A reduced the sensitivity of DLD-1 cells to CX258 treatment. Table S1: Differentially expressed mRNA after CX258 treatment [48].
