# Supplementary material for: Chalcone Derivative CX258 Suppresses Colorectal Cancer via Inhibiting the TOP2A/Wnt/β-Catenin Signaling
Source: Cells. 2023 Mar 31;12(7):1066. doi: 10.3390/cells12071066 (PMC10093515; doi:10.3390/cells12071066)
Supplement: Supplementary file 1 [file cells-12-01066-s001.zip › Supplementary File 1.pdf]

Supplementary File 1

Supplementary Materials For

Chalcone Derivative CX258 Suppresses Colorectal Cancer via Inhibiting the TOP2A/Wnt/β-Catenin Signaling

Xi Chen [1], Xiaocheng Lv [1], Lijie Gao [1], Jiawei Liu [1], Wei Wang [1], Lichao Guo [1], Mykhaylo S. Frasinyuk [2], Wen Zhang [3,4], David S. Watt [3,4], Chunming Liu [3,4,*] and Xifu Liu [1,*]

[1] Ministry of Education Key Laboratory of Molecular and Cellular Biology, Hebei Anti-Tumor Molecular Target Technology Innovation Center, College of Life Science, Hebei Normal University, Shijiazhuang 050024, China
[2] Institute of Bioorganic Chemistry and Petrochemistry, National Academy of Science of Ukraine, 02094 Kyiv, Ukraine
[3] Lucille Parker Markey Cancer Center, University of Kentucky, Lexington, KY 40536, USA
[4] Department of Molecular and Cellular Biochemistry, College of Medicine, University of Kentucky, Lexington, KY 40536, USA
* Correspondence: chunming.liu@uky.edu (C.L.); xfliu@hebtu.edu.cn (X.L.); Tel.: +1-8593234558 (C.L.); +86-13817831600 (X.L.)

This file includes:

1. Supplementary Section S1: Supplementary methods for chemical synthesis of chalcone derivatives.

2. Supplementary Section S2: Supplementary characterization of chalcone derivatives.

3. Supplementary Figure S1: Chalcone derivatives did not inhibited FOPFlash reporter activation.

4. Supplementary Figure S2: CX258 significantly inhibited TOPFlash activity.

5. Supplementary Figure S3: CX258 inhibited Wnt target gene transcription in LS174T and DLD-1 cells.

6. Supplementary Figure S4: Natural chalcones inhibited the proliferation of colon cancer cells.

7. Supplementary Figure S5: Knocking down of TOP2A reduced the sensitivity of DLD-1 cells to CX258 treatment.

Supplementary Section S1: Supplementary methods for chemical synthesis of chalcone derivatives

SUPPLEMENTARY METHODS

1. General considerations

Reagents and solvents were purchased from commercial suppliers and used without further purification. Chemicals and solvents were obtained from Energy Chemical (Shanghai, China), except for 3-hydroxyacetophenone (Shanghai Aladdin Biochemical Technology Co., Ltd., Shanghai, China), 4-hydroxyacetophenone (Shanghai Yi En Chemical Technology Co., Ltd., Shanghai, China), 2-quinolinecarboxaldehyde (Shanghai Macklin Biochemical Technology Co., Ltd., Shanghai, China), 4-quinolinecarboxaldehyde (Chengdu West Asia Chemical Co., Ltd., Chengdu, China) and 1,4-benzodioxane-6-carbaldehyde (Shanghai Kaiwei Chemical Technology Co., Ltd., Shanghai, China). ^{1}H NMR and ^{13}C NMR were studied using a AVIII500 nuclear magnetic resonance spectrometer (Brookhaven, Germany).

2. Experimental procedures for the preparation of compounds CX48, CX51, CX67, CX203, CX204, CX205, CX206 and CX258.

2.1. General Procedure A

To a 250 mL round-bottomed flask equipped with a magnetic stirrer was added sequentially *ca*. 10 mL of anhydrous ethanol, acetophenone (8 mmol), and benzaldehyde (8 mmol). The mixture was stirred for *ca*. 3 min at 0°C, and 1.84 mL of 50% aqueous potassium hydroxide was slowly added. Th mixture was stirred for 24 h at 25°C, and was poured into ice water. This basic mixture was neutralized by slowly adding a 20% acetic acid solution. The resulting precipitate was collected in a sintered glass funnel and washed with water. The crude product was recrystallized using anhydrous ethanol and dried under vacuum.

2.2. General procedure B

To a 250 mL round-bottomed flask equipped with a magnetic stirrer was added sequentially dichloromethane (16 mL), a chalcone analog (4 mmol) and triethylamine (4.8 mmol). The mixture was stirred at 0°C, and a dichloromethane solution of the chloride component (4.8 mmol, 16 mL) was slowly added. The reaction was stirred overnight at 25°C, and the mixture was concentrated to dryness using vacuum rotary evaporation. The crude product was purified by PE/EA (3/1) silica gel chromatography or was recrystallized from methanol.

2.3. General procedure C

To a 250 mL round-bottomed flask equipped with a magnetic stirrer was added sequentially DMSO (20 mL), benzaldehyde (5 mmol) and iodine (7.5 mmol). The

mixture was stirred for *ca*. 6 h at 110°C. The mixture was cooled, diluted with water and extracted with several portions of ethyl acetate. The combined organic layers were washed with brine, dried over anhydrous $MgSO_4$, and concentrated to dryness using vacuum rotary evaporation. The crude product was purified by PE/EA (10/1) silica gel chromatography.

2.4. Synthesis of 2-(3-(3,4-dimethoxyphenyl)acryloyl)-5-methoxyphenyl furan-2-carboxylate (CX48)

According to the general procedure A, the intermediate product of CX48 was prepared from 2'-hydroxy-4'-methoxyphenylacetophenone (1.33 g) and 3,4-dimethoxyphenyl formaldehyde (1.04 g). Using general procedure B, this product was treated with 2-furanylchloride to prepare the target compound CX48 as a bright yellow solid in a yield of 63% (1.66 g) after purification by column chromatography: mp 92-94°C. ^{1}H NMR (400 MHz, $CDCl_3$) δ 7.8 (d, J = 8.7 Hz, 1H), 7.57 (dd, J = 11, 8.3 Hz, 2H), 7.34 (d, J = 3.4 Hz, 1H), 7.06 (dd, J = 41, 8.7 Hz, 3H), 6.82 (dd, J = 5.3, 2.9 Hz, 3H), 6.5 (d, J = 1.8 Hz, 1H), 3.98–3.8 (m, 9H). ^{13}C NMR (126 MHz, $CDCl_3$) δ 189.44, 163.07, 156.41, 151.3, 150.1, 149.17, 147.23, 144.36, 143.77, 131.8, 127.8, 125.13, 123.33, 123.09, 119.91, 112.2, 112.1, 111.07, 110.97, 109.96, 108.95, 55.95, 55.88, 55.73. HR-MS (ESI-TOF) calculated for $C_{23}H_{20}O_7[M+H]^+$ 409.12873, found 409.12818.

2.5. Synthesis of 4-bromo-2-(3-(3,4-dimethoxyphenyl)acryloyl)phenyl thiophene-2-carboxylate (CX51)

According to general procedure A, the intermediate product leading to the title compound was prepared from 2-hydroxy-5-bromophenone (1.72 g) and 3,4-dimethoxybenzaldehyde (1.03 g). Using general procedure B, CX51 was prepared from this intermediate product (1.45 g) and 2-thiophenylchloride to obtain CX51 (1.03 g) in a yield of 91% as a white solid: mp 120-122°C. ^{1}H NMR (400 MHz, $CDCl_3$) δ 7.97–7.82 (m, 2H), 7.71 (dd, J = 8.6, 2.4 Hz, 3H), 7.27 (d, J = 8.6 Hz, 1H), 7.06 (ddd, J = 15.9, 7.6, 6.2 Hz, 4H), 6.86 (d, J = 8.3 Hz, 1H), 3.92 (d, J = 24.2 Hz, 6H). ^{13}C NMR (126 MHz, $CDCl_3$) δ 189.98, 159.91, 151.69, 149.15, 147.23, 146.45, 135.31, 134.92, 134.56, 134.16, 132.58, 131.77, 128.13, 127.23, 125.17, 123.72, 122.93, 119.45, 110.96, 109.72, 55.99, 55.85. HR-MS (ESI-TOF) calculated for $C_{22}H_{17}BrO_5S[M+H]^+$ 473.00583, $[M+2H]^{2+}$ 475.02148, found 473.00528,475.00324.

2.6. Synthesis of 4-bromo-2-(3-(3,4-dimethoxyphenyl)acryloyl)phenyl thiophene-2-carboxylate (CX67)

According to general procedure A, the title compound was prepared from 2-hydroxy-5-methylthiophenone (1.2 g) and o-chlorobenzaldehyde (1.11 g). Using 4-DMAP as a catalyst and following general procedure B, CX67 was prepared from the intermediate product of the title compound and 4-morpholinoyl chloride. After purification by column chromatography, the yield was 78% (1.2 g) of a white solid: mp 110-112°C. ^{1}H NMR (400 MHz, $CDCl_3$) δ 7.99 (d, J = 16 Hz, 1H), 7.68 (dd, J = 7.5, 1.8 Hz, 1H), 7.52–7.41 (m, 2H), 7.37–7.26 (m, 3H), 7.12 (dd, J = 12.1, 8.1 Hz, 2H),

3.54 (t, J = 21.9 Hz, 8H), 2.4 (s, 3H). ^{13}C NMR (126 MHz, $CDCl_3$) δ 191.52, 153.18, 147.14, 140.04, 135.51, 135.46, 133.25, 132.94, 131.99, 131.35, 130.41, 130.15, 128.09, 127.61, 127.13, 123.34, 66.53, 66.32, 44.89, 44.17, 20.8. HR-MS (ESI-TOF) calculated for $C_{21}H_{20}ClNO_4$[M +H]$^+$ 386.11591, found 386.11536.

2.7. Synthesis of 1-(4-hydroxyphenyl)-3-(quinolin-4-yl)prop-2-en-1-one (CX203)

The title compound was prepared from 4-hydroxyacetophenone (1.36 g) and 4-quinolinecarboxaldehyde (1.57 g) according to general procedure A to afford, after purification by recrystallization from absolute ethanol, compound CX203 in 91% yield (2.5 g) as a yellow solid: mp 244-246°C. ^{1}H NMR (400 MHz, DMSO) δ 10.55 (s, 1H), 9.01 (d, J = 4.5 Hz, 1H), 8.43 (d, J = 15.4 Hz, 1H), 8.34 (d, J = 8.4 Hz, 1H), 8.22–8.06 (m, 5H), 7.85 (t, J = 7.6 Hz, 1H), 7.73 (t, J = 7.6 Hz, 1H), 6.96 (d, J = 8.6 Hz, 2H). ^{13}C NMR (126 MHz, DMSO) δ 187.34, 163.06, 150.74, 148.69, 140.21, 136.51, 131.94, 130.17, 130.1, 129.15, 129.03, 127.79, 126.19, 123.92, 119.04, 116. HR-MS (ESI-TOF) calculated for $C_{18}H_{13}NO_2$[M+H]$^+$ 274.0868, found 274.08626.

2.8. Synthesis of 1-(4-hydroxyphenyl)-3-(quinolin-2-yl)prop-2-en-1-one (CX204)

The title compound was prepared from 4-hydroxyacetophenone (1.36 g) and 2-quinolinecarboxaldehyde (1.57 g) according to general procedure A to afford, after purification by recrystallization from absolute ethanol, compound CX204 in 85% yield (2.333 g) as a yellow solid: mp 235-237°C. ^{1}H NMR (400 MHz, DMSO) δ 10.53 (s, 1H), 8.47 (d, J = 8.5 Hz, 1 H), 8.31 (d, J = 15.6 Hz, 1H), 8.2 (d, J = 8.5 Hz, 1H), 8.08 (dd, J = 22.1, 10.3 Hz, 3H), 8.01 (d, J = 8.1 Hz, 1H), 7.8 (dd, J = 15.1, 5.8 Hz, 2H), 7.65 (t, J = 7.4 Hz, 1H), 6.94 (d, J = 8.3 Hz, 2H). ^{13}C NMR (126 MHz, DMSO) δ 187.66, 162.99, 154.3, 148.08, 142.73, 137.34, 131.83, 130.59, 129.66, 129.25, 128.31, 128.22, 127.8, 127.56, 121.29, 116.01. HR-MS (ESI-TOF) calculated for $C_{18}H_{13}NO_2$[M +H]$^+$ 274.08680, found 274.08626.

2.9. Synthesis of 1-(3-hydroxyphenyl)-3-(quinolin-2-yl)prop-2-en-1-one (CX205)

The title compound was prepared from 3-hydroxyacetophenone (1.36 g) and 2-quinolinecarboxaldehyde (1.573 g) according to general procedure A to afford, after purification by recrystallization from absolute ethanol, compound CX205 in 73% yield (2 g) as a yellow solid: mp 232-234°C. ^{1}H NMR (500 MHz, DMSO) δ 9.89 (s, 1H), 8.83 (d, J = 8.6 Hz, 1H), 8.56–8.41 (m, 2H), 8.32 (d, J = 8.5 Hz, 1H), 8.17 (d, J = 8 Hz, 1H), 7.97 (dd, J = 15.1, 8.6 Hz, 2H), 7.8 (t, J = 7.3 Hz, 1H), 7.72 (d, J = 7.9 Hz, 1H), 7.6–7.52 (m, 1H), 7.43 (t, J = 7.9 Hz, 1H), 7.22–7.05 (m, 1H). ^{13}C NMR (126 MHz, DMSO) δ 189.91, 158.28, 158.13, 154, 148.09, 143.64, 139.1, 137.42, 130.64, 130.45, 129.7, 128.32, 127.89, 127.64, 121.51, 121.1, 121, 120.16, 120.12, 115.17, 115.08. HR-MS (ESI-TOF) calculated for $C_{18}H_{13}NO_2$[M+H]$^+$ 274.0868, found 274.08626.

2.10. Synthesis of 1-(3-hydroxyphenyl)-3-(6-methoxyquinolin-2-yl)prop-2-en-1-one (CX206)

According to general procedure C, the intermediate product of the title compound was prepared from 6-methylquinoline-2-formaldehyde (0.856 g) and iodine (1.9 g).

According to general procedure A, CX206 was prepared from the intermediate of the title compound and 1,4-benzodioxane-6-formaldehyde (1.09 g) in 35% (0.85 g) yield as a reddish-brown solid: mp 204-206℃. ^{1}H NMR (400 MHz, DMSO) δ 9.89 (s, 1H), 8.36 (d, J = 8.6 Hz, 1H), 8.17 (dd, J = 17, 12.1 Hz, 2H), 8 (d, J = 9.1 Hz, 1H), 7.81 (d, J = 15.7 Hz, 1H), 7.65 (d, J = 7.7 Hz, 1H), 7.46 (ddd, J = 19.5, 9.6, 7 Hz, 4H), 7.11 (dd, J = 8.1, 2 Hz, 1H), 3.94 (s, 3H). ^{13}C NMR (126 MHz, DMSO) δ 189.8, 158.52, 158.25, 151.42, 144.18, 143.96, 139.19, 135.99, 131.26, 130.44, 129.67, 126.22, 123.25, 121.89, 120.99, 120.06, 115.09, 106.08, 56.04. HR-MS (ESI-TOF) calculated for $C_{19}H_{15}NO_3[M+H]^+$ 274.0868, found 306.11247.

2.11. Synthesis of 1-(5-chloro-2-methoxyphenyl)-3-(2,3-dihydrobenzo[*b*][1,4]dioxin-6-yl)prop-2-en-1-one (CX258) [48]

The title compound was prepared from 1-(5-chloro-2-methoxyphenyl)ethan-1-one (1.47 g) and 1,4-benzodioxane-6-carbaldehyde (1.3 g) according to general procedure A to afford, after purification by recrystallization from absolute ethanol, CX258 in 81% yield (2.141 g) as a white solid: mp 115-117℃. ^{1}H NMR (400 MHz, $CDCl_3$) δ 7.53 (dd, J = 15.8, 9.3 Hz, 2H), 7.4 (d, J = 8.8 Hz, 1H), 7.19–7.07 (m, 3H), 6.9 (dd, J = 19.6, 8.6 Hz, 2H), 4.29 (q, J = 5 Hz, 4H), 3.87 (s, 3H). ^{13}C NMR (126 MHz, $CDCl_3$) δ 191.44, 156.51, 145.98, 143.98, 143.72, 132.11, 130.65, 129.88, 128.51, 125.93, 124.8, 122.73, 117.75, 116.99, 113.05, 64.59, 64.21, 56.13. HR-MS (ESI-TOF) calculated for $C_{18}H_{15}ClO_4[M+H]^+$ 331.07371, found 331.07316.

Supplementary Section S2:

Supplementary characterization of chalcone derivatives

1. NMR spectra of compounds

2-(3-(3,4-dimethoxyphenyl)acryloyl)-5-methoxyphenyl furan-2-carboxylat -e (CX48):

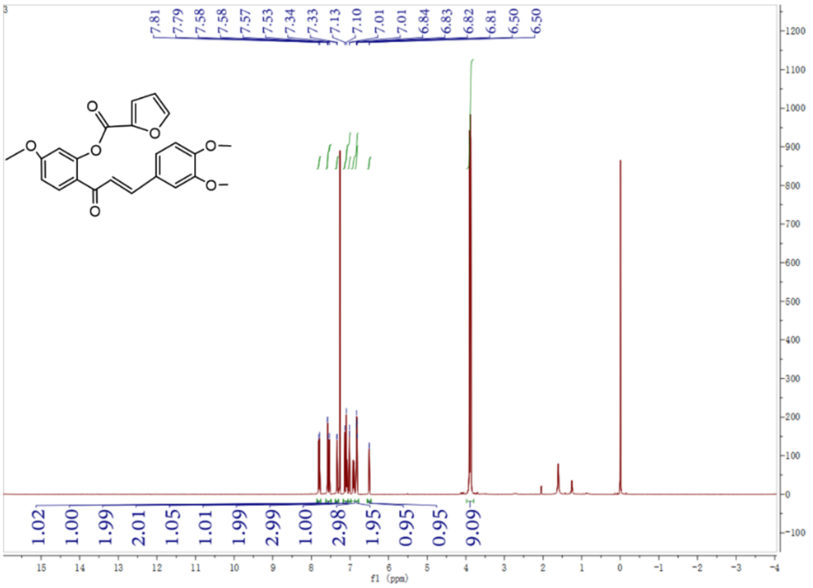

^{1}H NMR (400 MHz, $CDCl_3$)

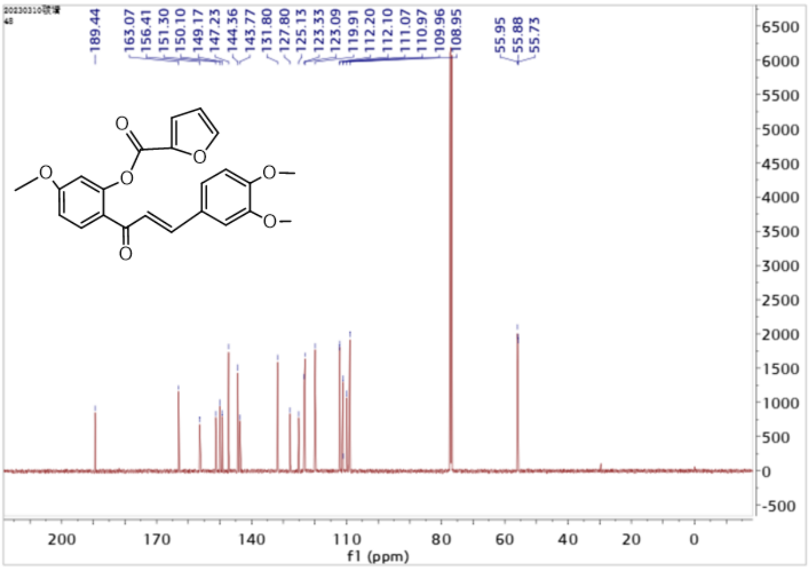

^{13}C NMR (126 MHz, $CDCl_3$)

4-bromo-2-(3-(3,4-dimethoxyphenyl)acryloyl)phenyl thiophene-2-carboxylate(CX51)

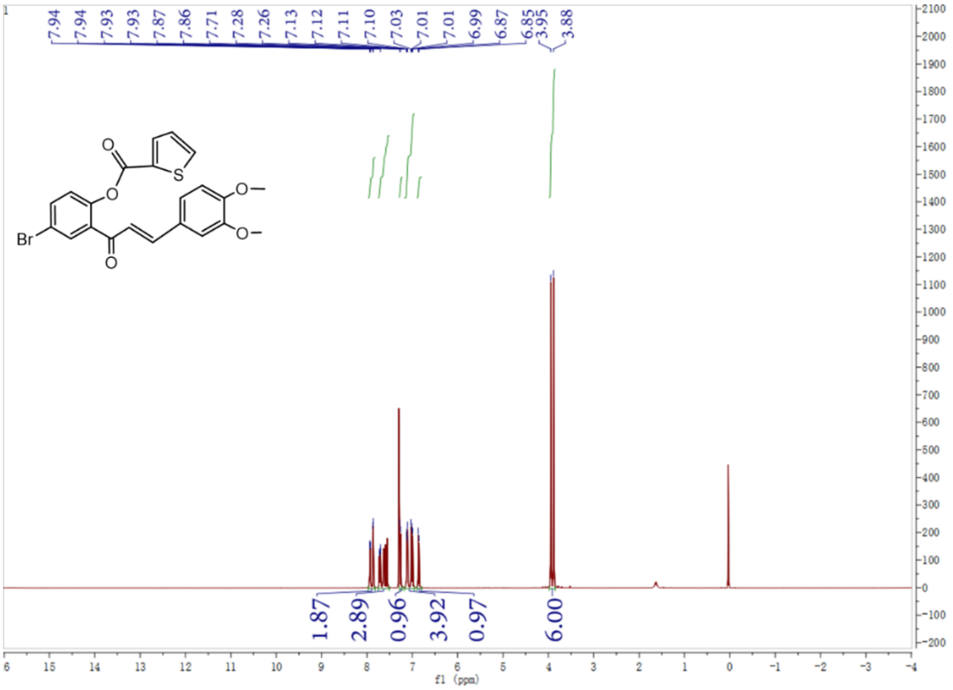

^{1}H NMR (400 MHz, $CDCl_3$)

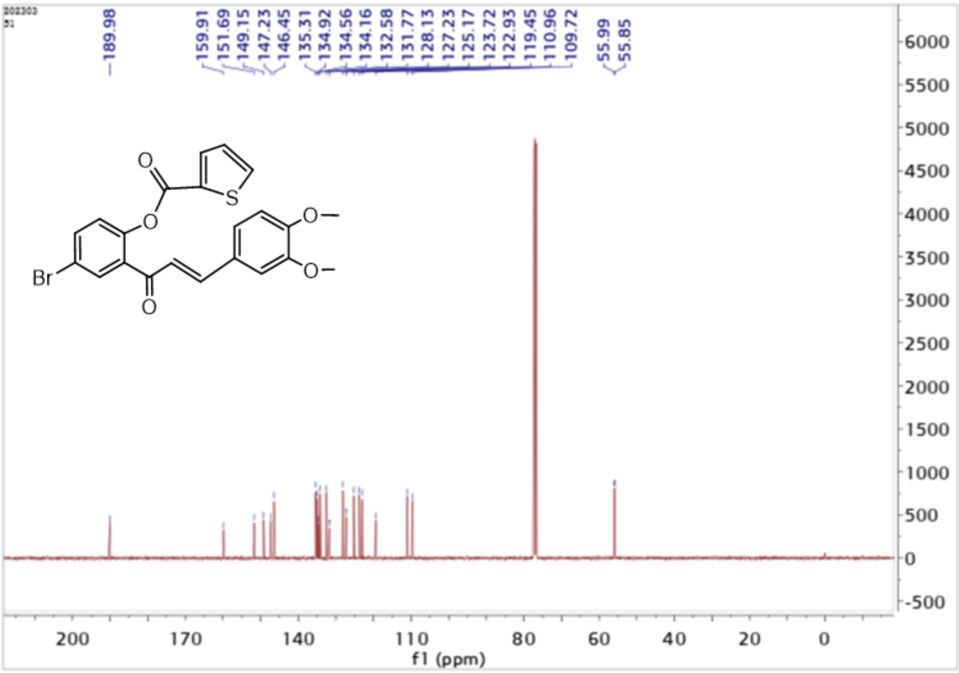

^{13}C NMR (126 MHz, $CDCl_3$)

4-bromo-2-(3-(3,4-dimethoxyphenyl)acryloyl)phenyl thiophene-2-carboxy-late(CX67):

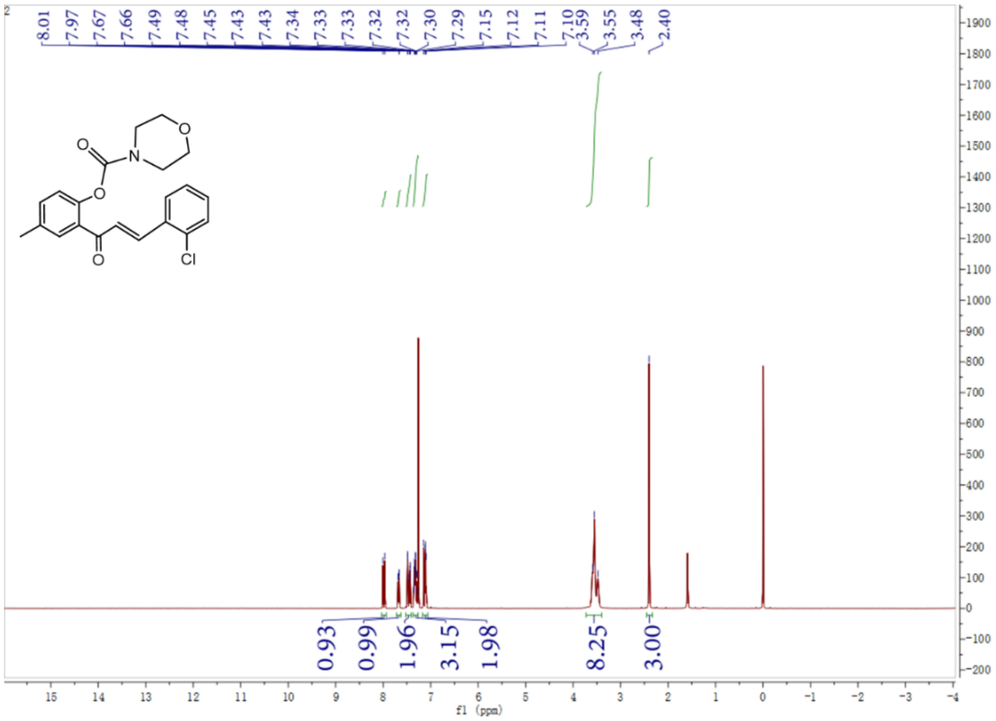

^{1}H NMR (400 MHz, $CDCl_3$)

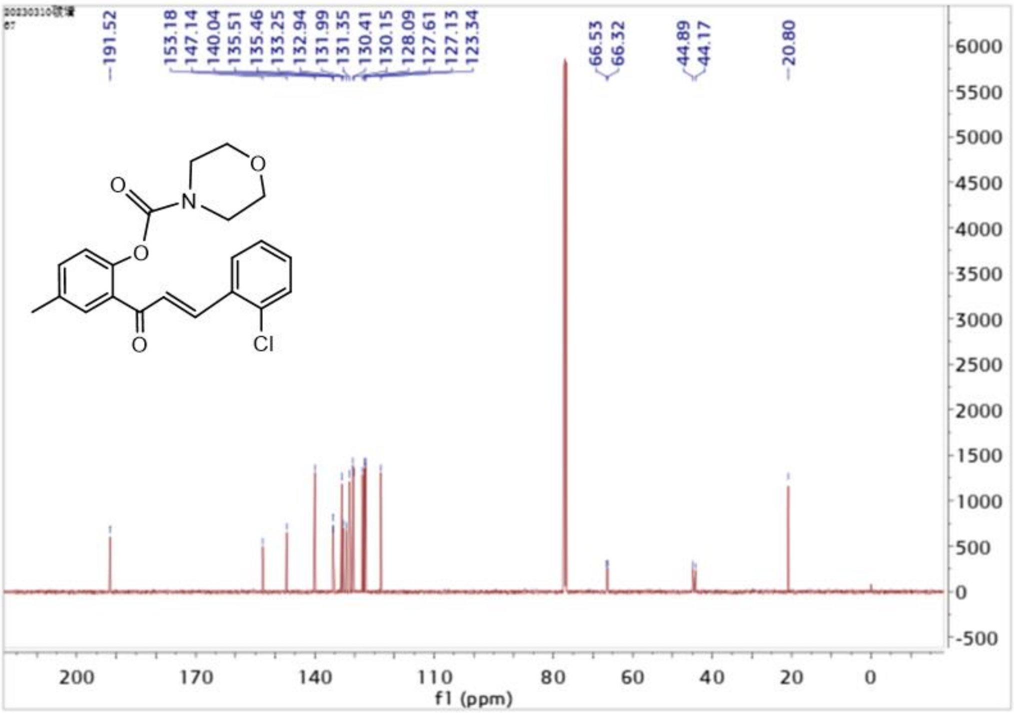

^{13}C NMR (126 MHz, $CDCl_3$)

1-(4-hydroxyphenyl)-3-(quinolin-4-yl)prop-2-en-1-one(CX203):

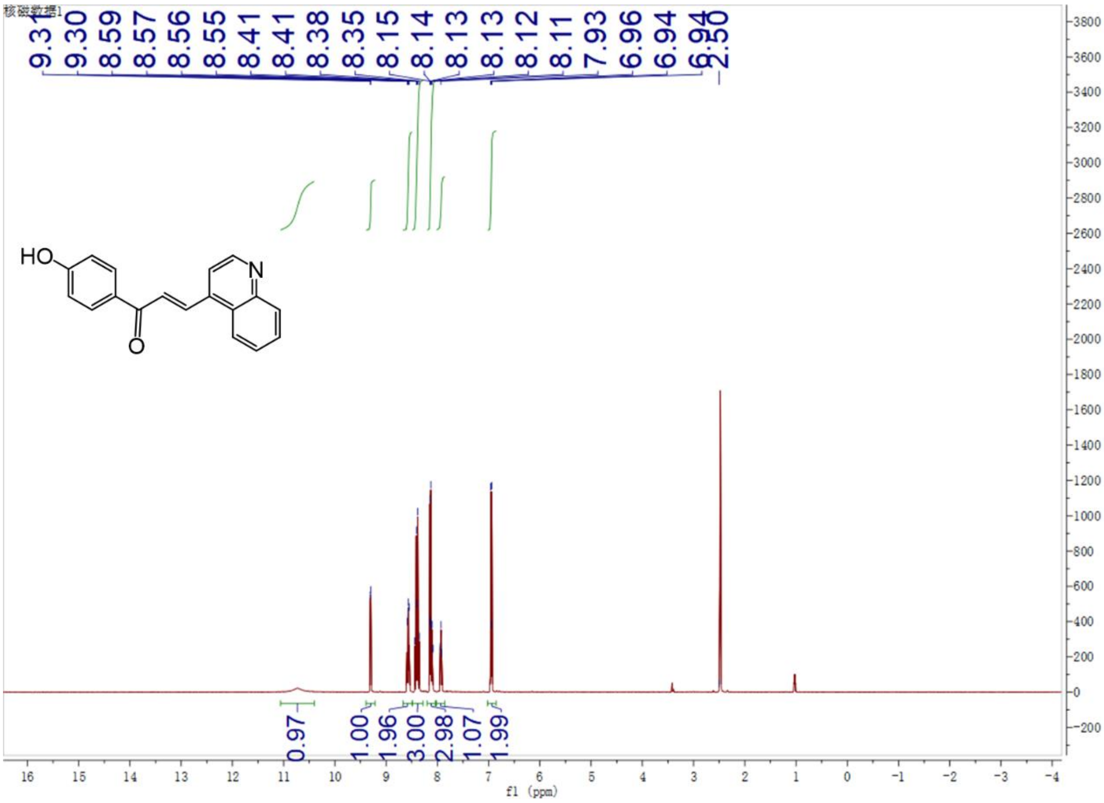

^{1}H NMR (400 MHz, DMSO)

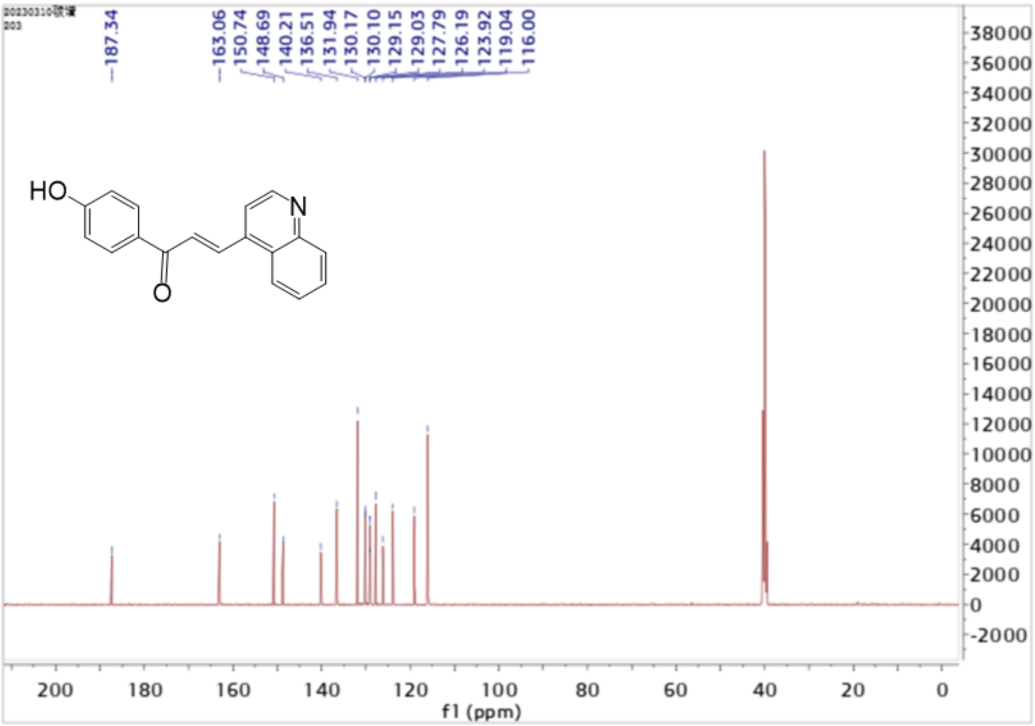

^{13}C NMR (126 MHz, DMSO)

1-(4-hydroxyphenyl)-3-(quinolin-2-yl)prop-2-en-1-one(CX204):

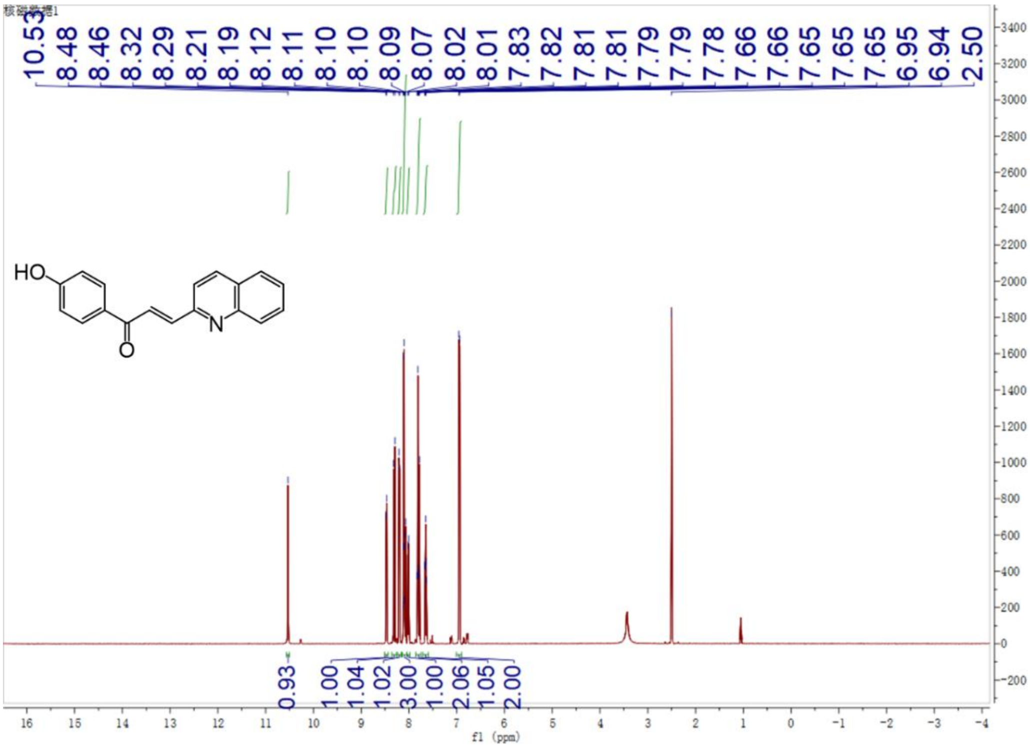

^{1}H NMR (400 MHz, DMSO)

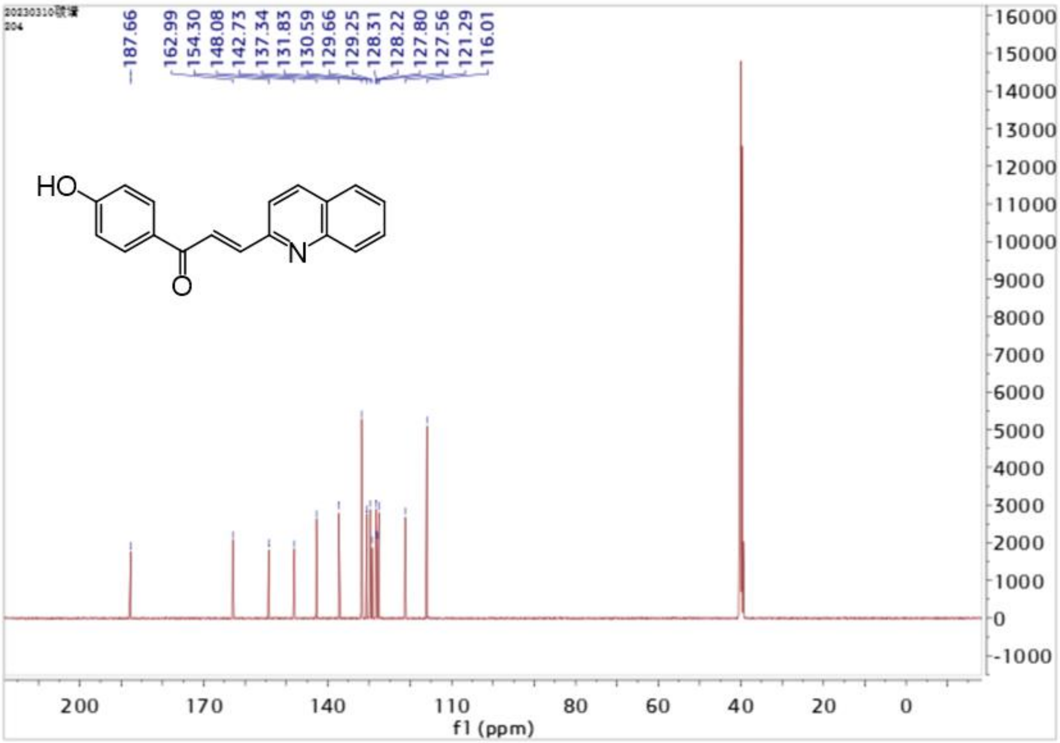

^{13}C NMR (126 MHz, DMSO)

1-(3-hydroxyphenyl)-3-(quinolin-2-yl)prop-2-en-1-one(CX205):

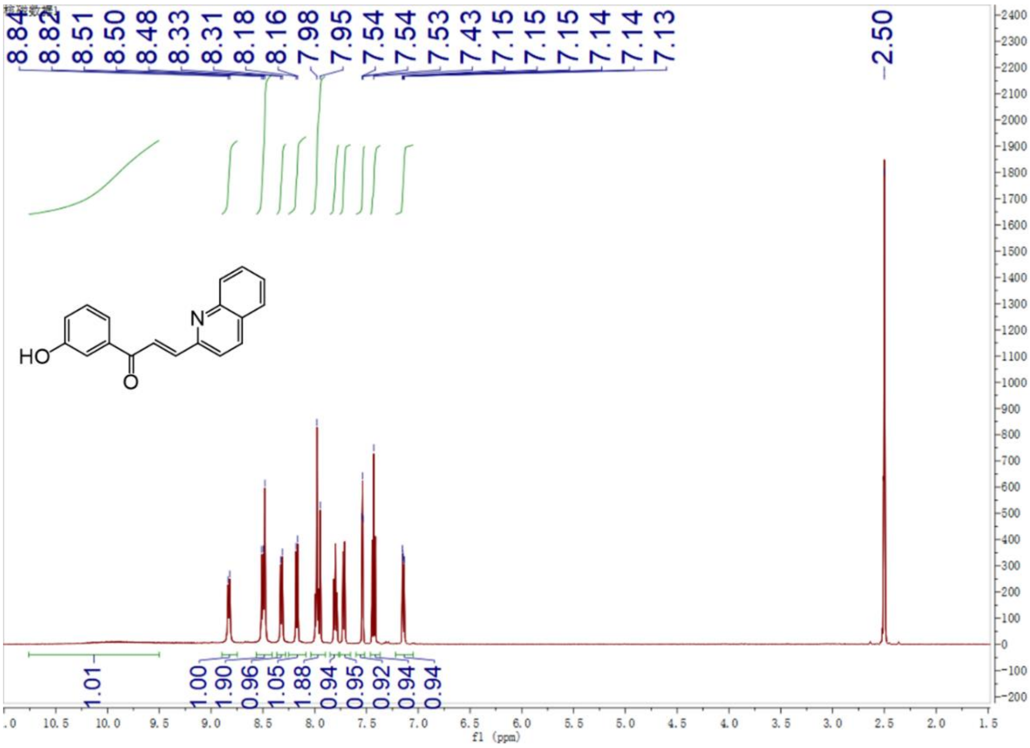

^{1}H NMR (500 MHz, DMSO)

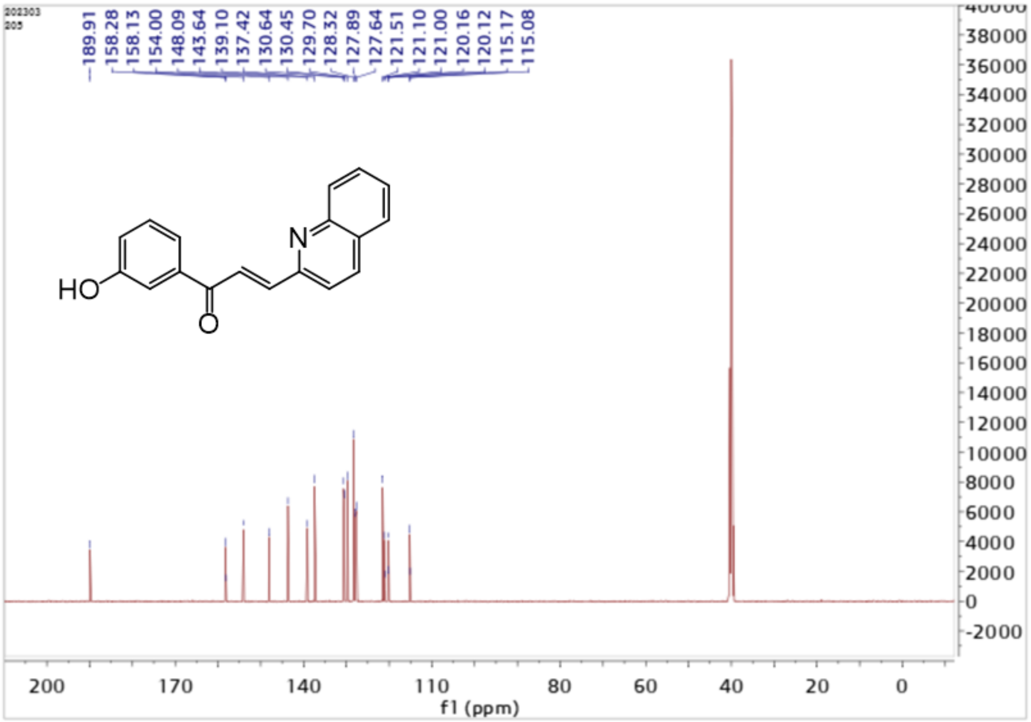

^{13}C NMR (126 MHz, DMSO)

1-(3-hydroxyphenyl)-3-(6-methoxyquinolin-2-yl)prop-2-en-1-one(CX206):

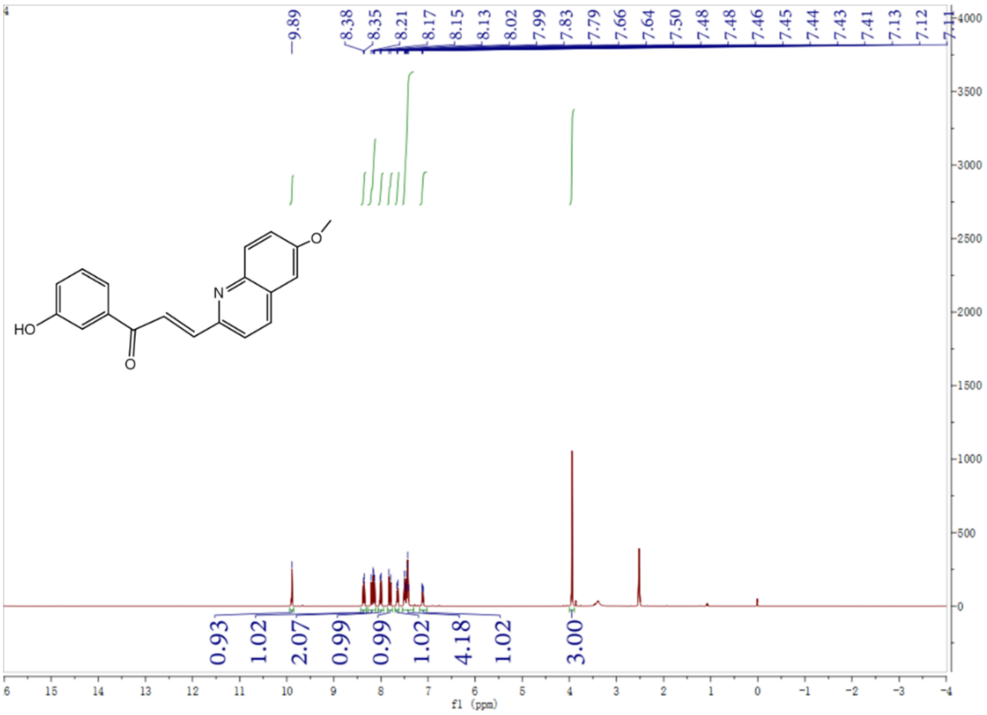

^{1}H NMR (400 MHz, DMSO)

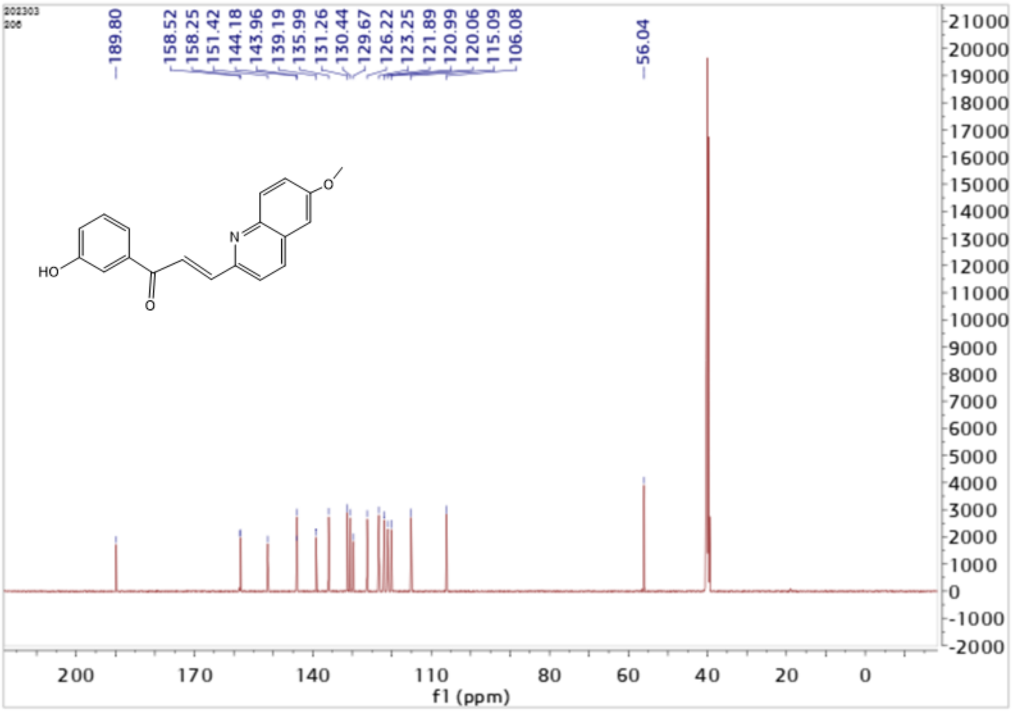

^{13}C NMR (126 MHz, DMSO)

1-(5-chloro-2-methoxyphenyl)-3-(2,3-dihydrobenzo[b][1,4]dioxin-6-yl)pr - op-2-en-1-one (CX258):

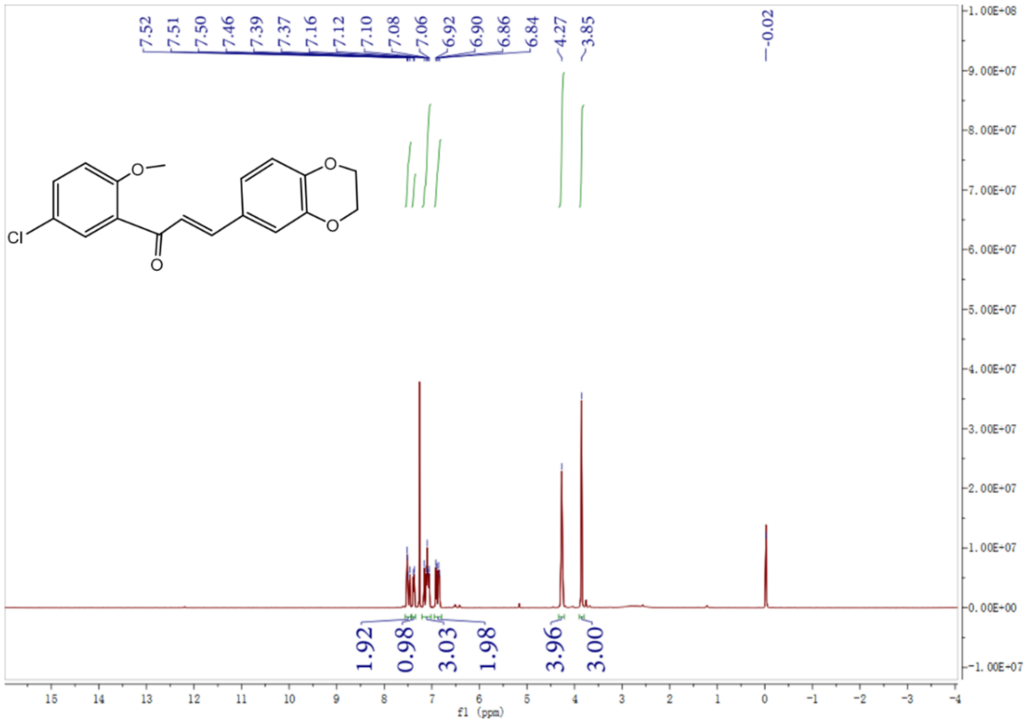

^{1}H NMR (400 MHz, $CDCl_3$)

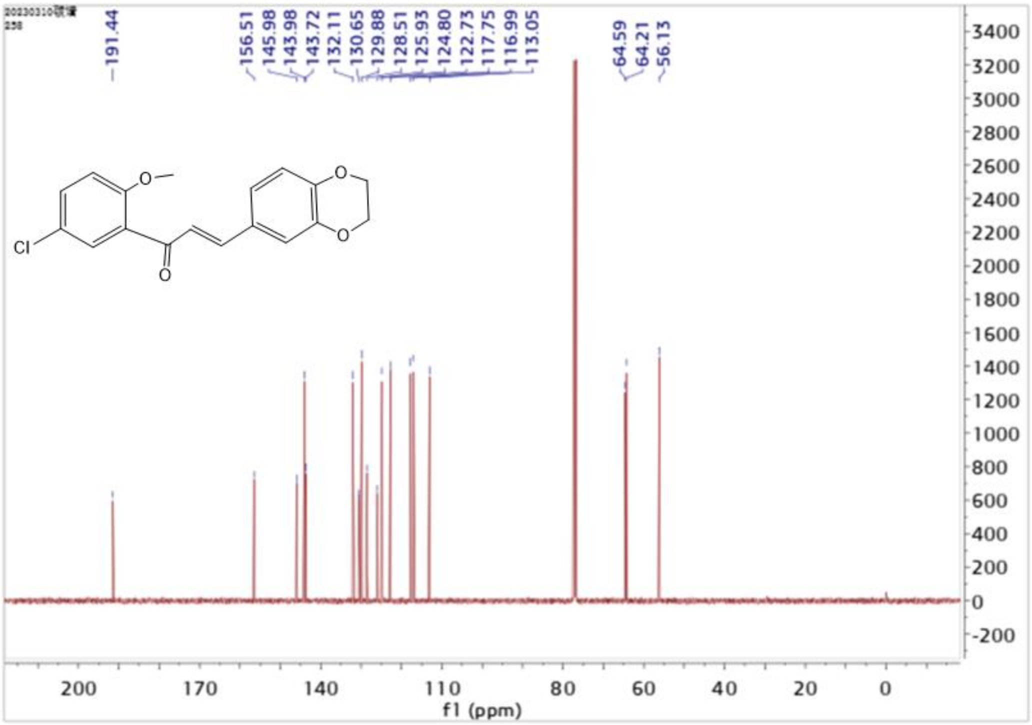

^{13}C NMR (126 MHz, $CDCl_3$)

2. High Resolution Mass Spectrometry spectra of compounds

CX48:

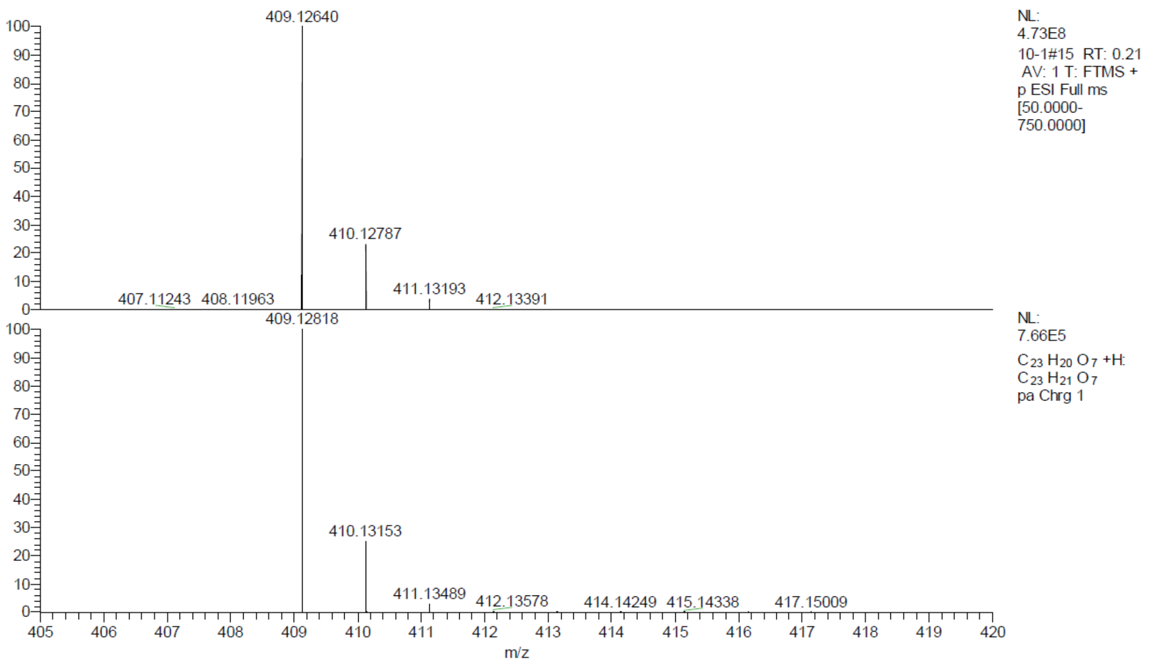

CX51:

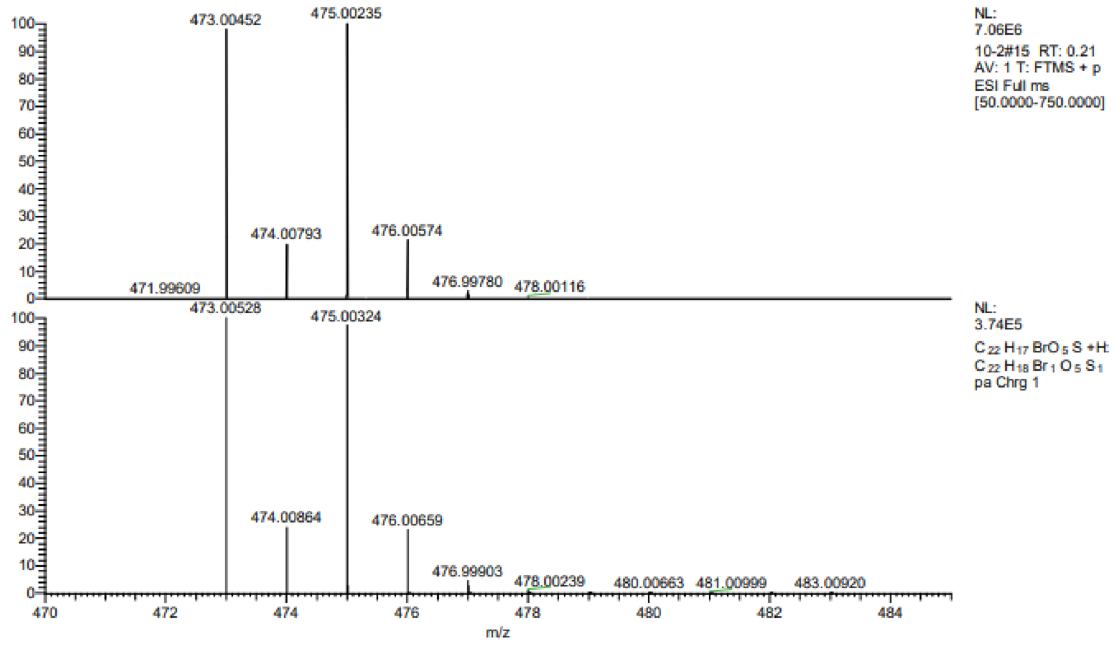

CX67:

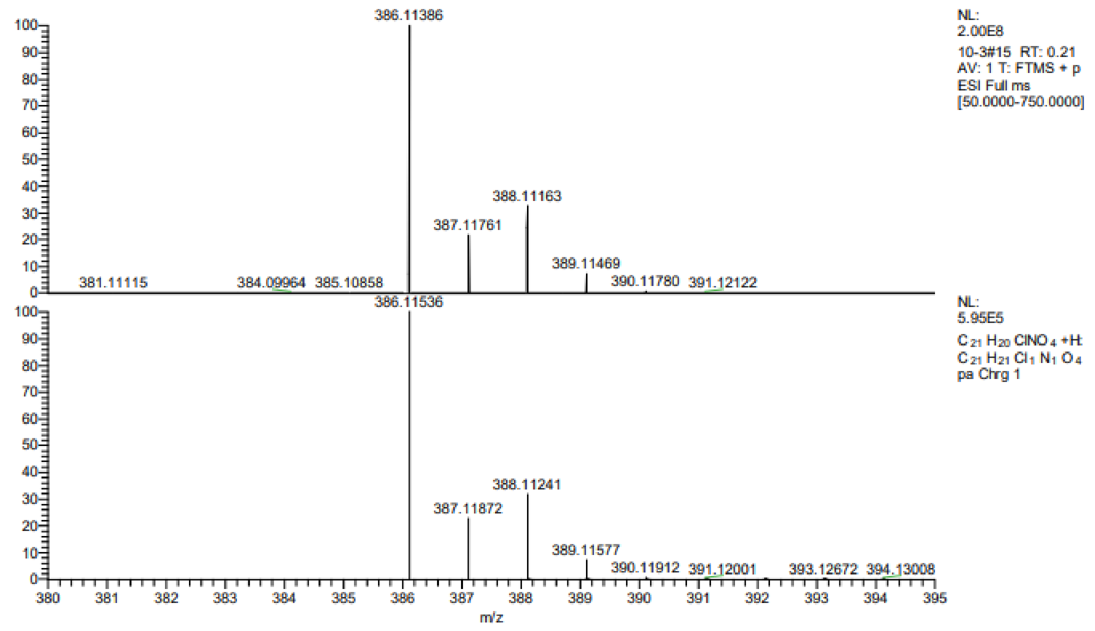
386.11386
388.11163
387.11761
389.11469
381.11115
384.09964
385.10858
390.11780
391.12122
NL: 2.00E8
10-3#15 RT: 0.21 AV: 1 T: FTMS + p ESI Full ms [50.0000-750.0000]
386.11536
388.11241
387.11872
389.11577
390.11912
391.12001
393.12672
394.13008
NL: 5.95E5
C 21 H20 ClNO 4 +H: C 21 H21 Cl1 N1 O 4 pa Chrg 1
m/z

CX203:

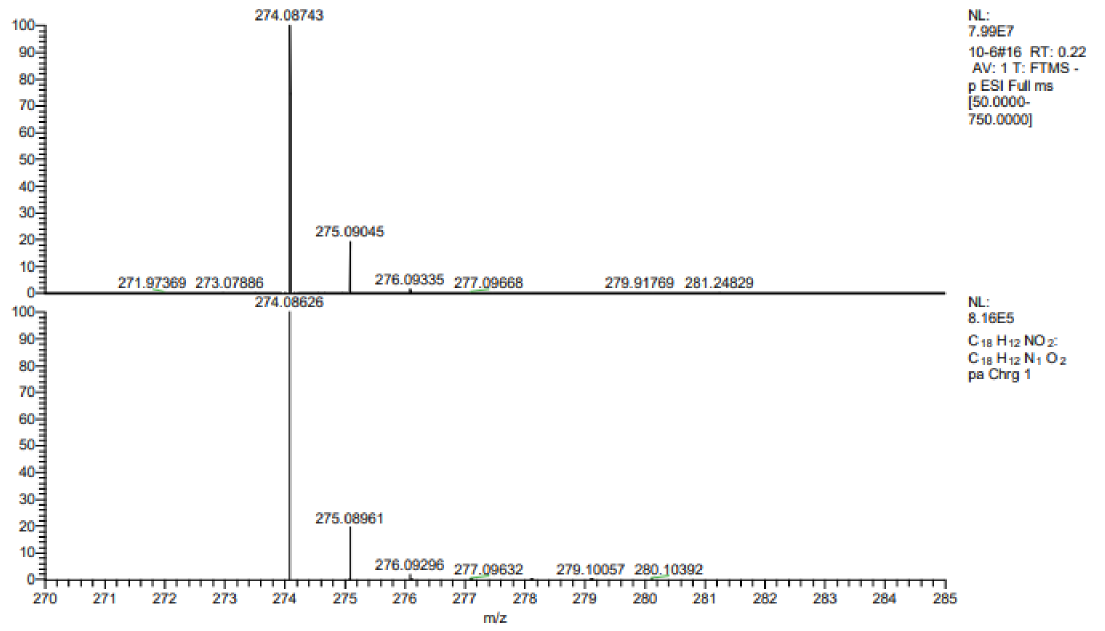
274.08743
275.09045
271.97369
273.07886
276.09335
277.09668
279.91769
281.24829
NL: 7.99E7
10-6#16 RT: 0.22 AV: 1 T: FTMS - p ESI Full ms [50.0000-750.0000]
274.08626
275.08961
276.09296
277.09632
279.10057
280.10392
NL: 8.16E5
C18 H12 NO 2: C18 H12 N1 O 2 pa Chrg 1
m/z

CX204:

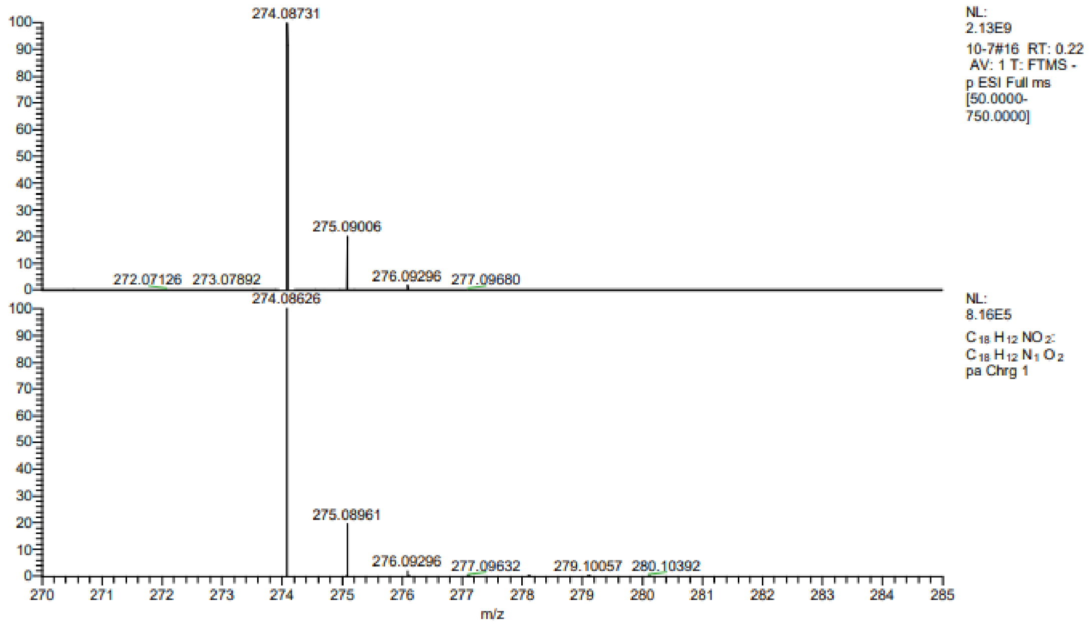
274.08731
275.09006
272.07126 273.07892
276.09296 277.09680
NL: 2.13E9
10-7#16 RT: 0.22 AV: 1 T: FTMS - p ESI Full ms [50.0000-750.0000]
274.08626
275.08961
276.09296 277.09632 279.10057 280.10392
NL: 8.16E5
C 18 H 12 NO 2: C 18 H 12 N 1 O 2 pa Chrg 1
m/z

CX205:

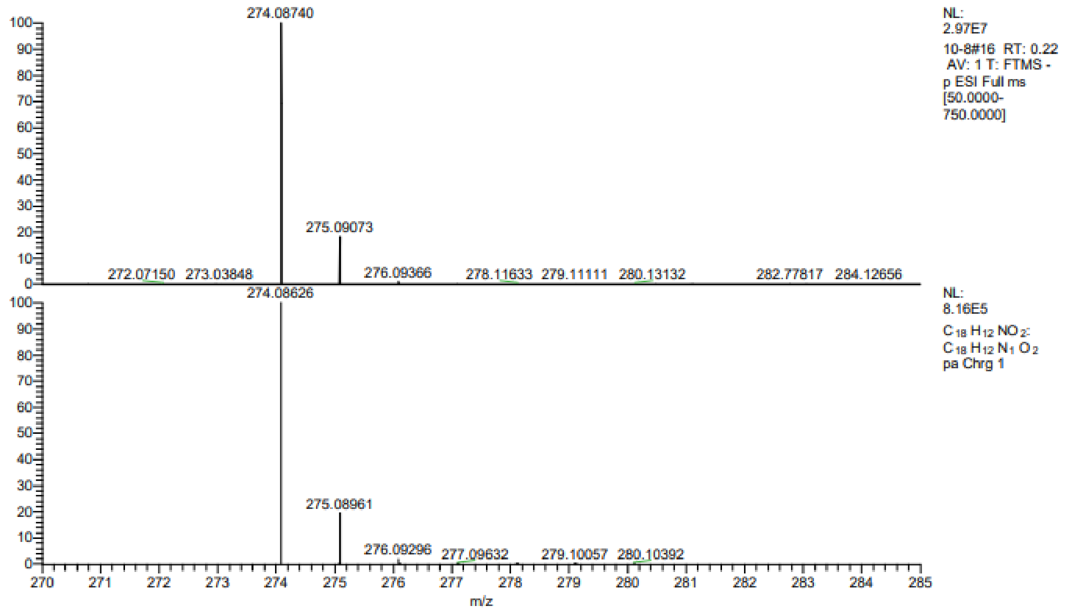
274.08740
275.09073
272.07150 273.03848
276.09366 278.11633 279.11111 280.13132 282.77817 284.12656
NL: 2.97E7
10-8#16 RT: 0.22 AV: 1 T: FTMS - p ESI Full ms [50.0000-750.0000]
274.08626
275.08961
276.09296 277.09632 279.10057 280.10392
NL: 8.16E5
C 18 H 12 NO 2: C 18 H 12 N 1 O 2 pa Chrg 1
m/z

CX206:

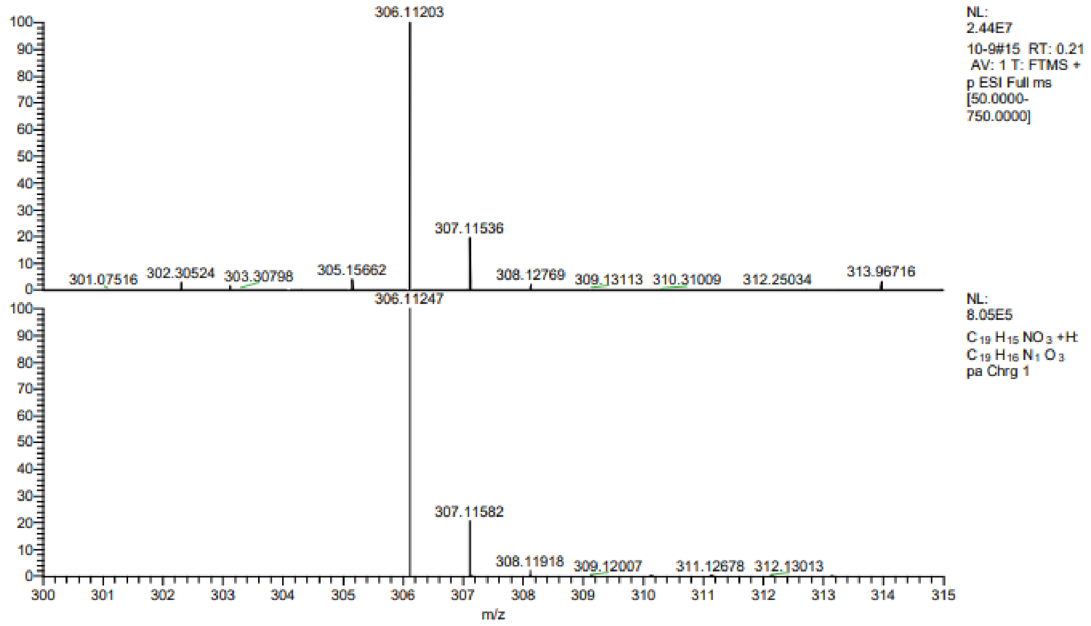

306.11203
307.11536
301.07516
302.30524
303.30798
305.15662
308.12769
309.13113
310.31009
312.25034
313.96716
NL:
2.44E7
10-9#15 RT: 0.21
AV: 1 T: FTMS +
p ESI Full ms
[50.0000-
750.0000]
306.11247
307.11582
308.11918
309.12007
311.12678
312.13013
NL:
8.05E5
C 19 H 15 NO 3 +H:
C 19 H 16 N 1 O 3
pa Chrg 1
m/z

CX258

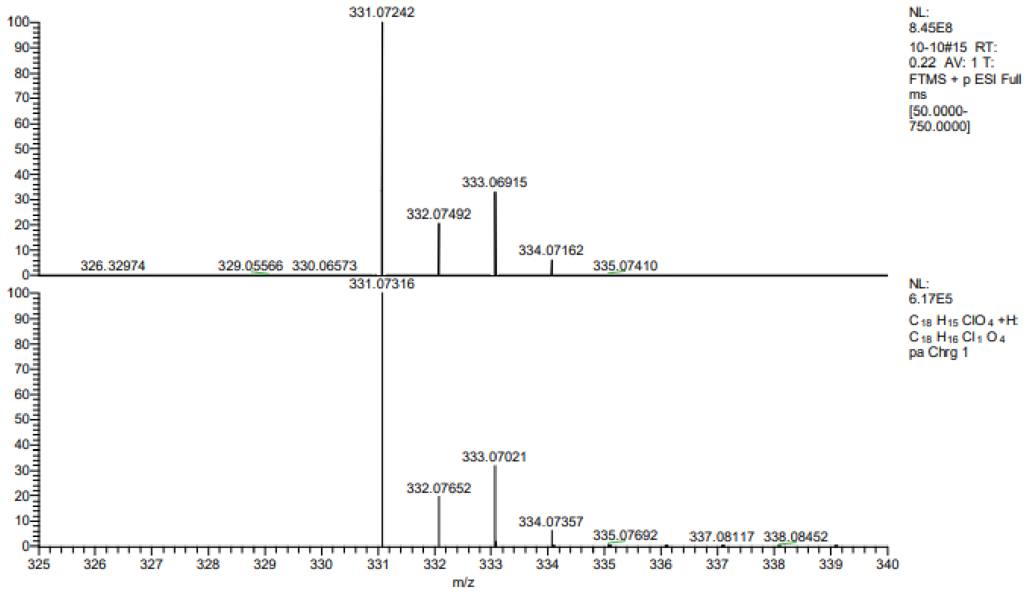

331.07242
333.06915
332.07492
334.07162
326.32974
329.05566
330.06573
335.07410
NL:
8.45E8
10-10#15 RT:
0.22 AV: 1 T:
FTMS + p ESI Full
ms
[50.0000-
750.0000]
331.07316
333.07021
332.07652
334.07357
335.07692
337.08117
338.08452
NL:
6.17E5
C 18 H 15 ClO 4 +H:
C 18 H 16 Cl 1 O 4
pa Chrg 1
m/z

SUPPLEMENTARY FIGURES WITH LEGENDS:

Supplementary Figure S1: Chalcone derivatives did not inhibited FOPFlash reporter activation

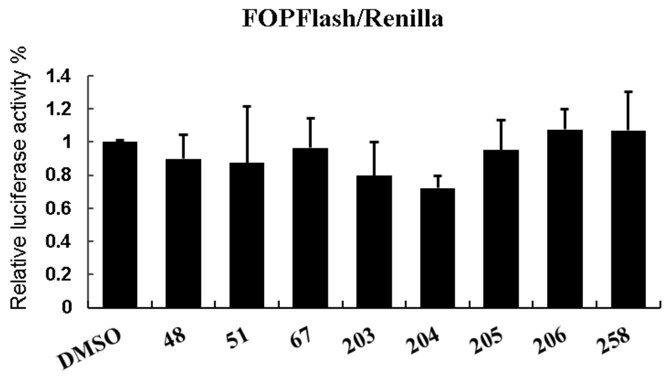

Figure S1. Chalcone derivatives did not inhibited FOPFlash reporter activation. FOPFlash was used as a negative control to verify the specificity of chalcone derivatives for Wnt signaling.

Supplementary Figure S2: CX258 significantly inhibited TOPFlash activity

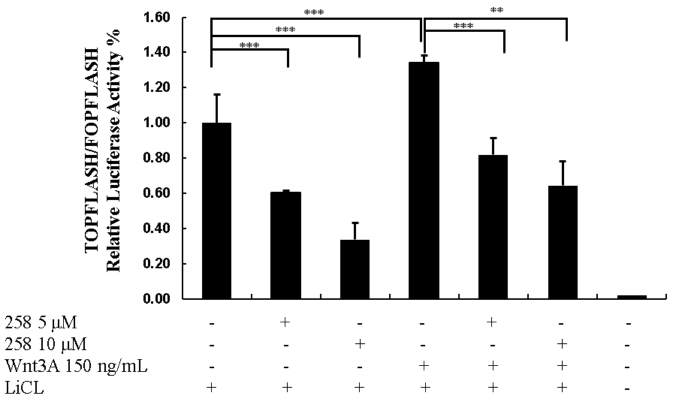

Figure S2. CX258 significantly inhibited TOPFlash activity. TOPFlash or FOPFlash (400 ng/well) plasmid was transfected into HEK-293T cells. The cells were treated with Wnt3A 150 ng/mL to enhance Wnt signaling. ** $p < 0.01$, *** $p < 0.001$.

Supplementary Figure S3: CX258 inhibited Wnt target gene transcription in LS174T and DLD-1 cells

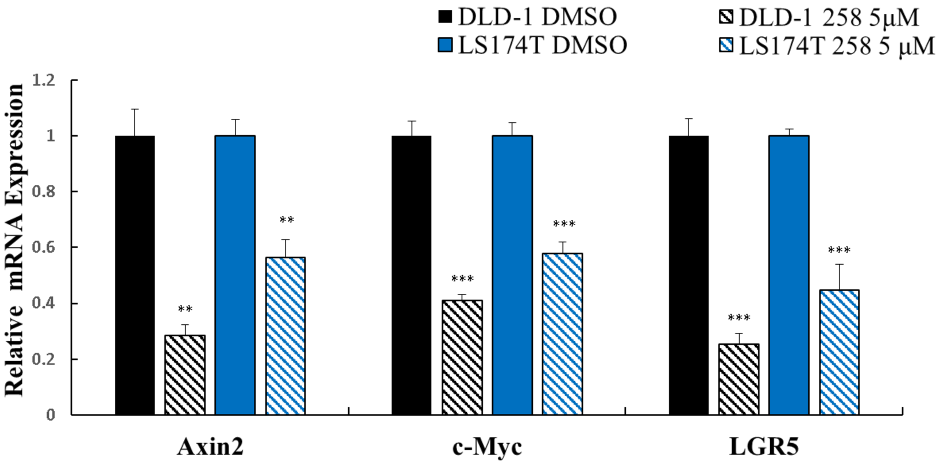

Figure S3. CX258 inhibited Wnt target gene transcription in LS174T and DLD-1 cells. LS174T and DLD-1 cells were treated with DMSO or CX258. The mRNA levels of Wnt target genes, Axin2, c-Myc and LGR5, were analyzed by qRT-PCR. ** $p < 0.01$, *** $p < 0.001$ vs. DMSO.

Supplementary Figure S4: Natural chalcones inhibited the proliferation of colon cancer cells

A

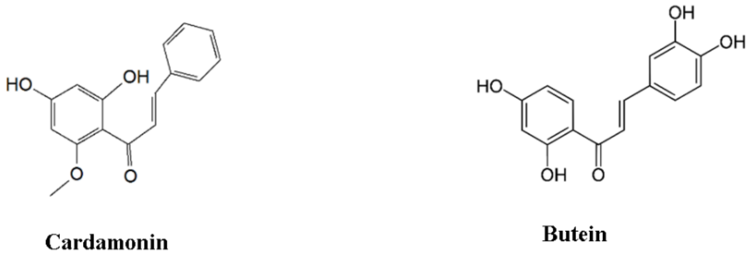

B

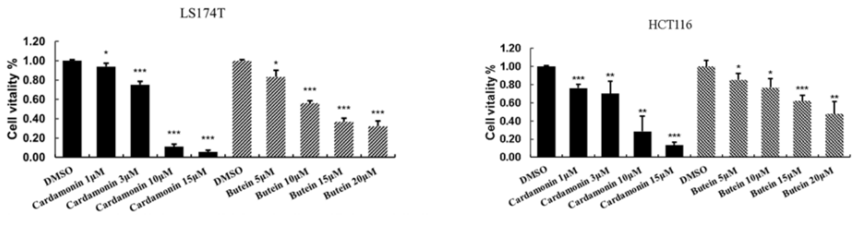

Figure S4. Natural chalcones inhibited the proliferation of colon cancer cells. A. Chemical structures of natural chalcones, cardamonin and butein. B. Inhibition effects of different concentrations of cardamonin and butein on the proliferation of CRC cells. Data are represented as means ± SD. * $p < 0.05$, ** $p < 0.01$, *** $p < 0.001$.

Supplementary Figure S5: Knocking down of TOP2A reduced the sensitivity of DLD-1 cells to CX258 treatment

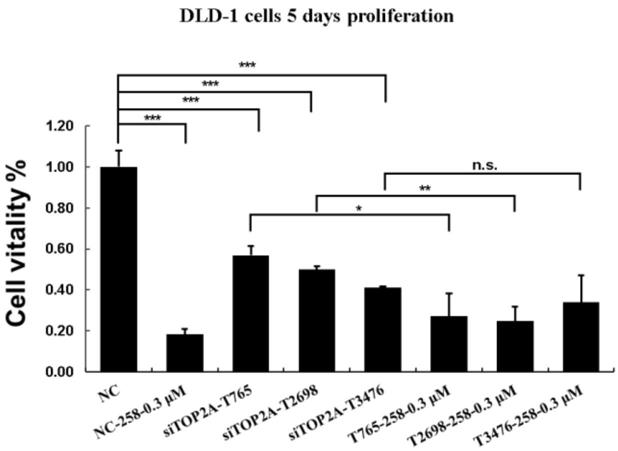

Figure S5. Knocking down of TOP2A reduced the sensitivity of DLD-1 cells to CX258 treatment. DLD-1 cells were transfected with TOP2A siRNAs and then treated with CX258. Cell proliferation was analyzed after 5 days. Data are presented as the mean ± SD. * $p < 0.05$, ** $p < 0.01$, *** $p < 0.001$.
